# From Infection to Oncology: AI-Assisted Drug Repurposing Uncovers Anti-Infective and Excipient-Class Compounds as PPAR-α-Linked Anticancer Agents

**DOI:** 10.3390/cancers18193231

**Published:** 2026-10-08

**Authors:** Shoeb Ikhlas, Amani Alsharidah, Reem A. Alkhodier, Sergio Senar, Mohamed Boudjelal

**Affiliations:** 1 King Abdullah International Medical Research Center (KAIMRC), Riyadh 11481, Saudi Arabia; ikhlass@kaimrc.med.sa (S.I.); khodierr@ksau-hs.edu.sa (R.A.A.); 2 King Saud Bin Abdulaziz University for Health Sciences, Riyadh 11481, Saudi Arabia; 3 Ministry of National Guard Health Affairs (MNGHA), Riyadh 11481, Saudi Arabia; 4 College of Science, King Saud University, Riyadh 11459, Saudi Arabia; 441203711@student.ksu.edu.sa; 5 Department of Pharmaceutical Sciences, College of Pharmacy, King Saud Bin Abdulaziz University for Health Sciences, Riyadh 11481, Saudi Arabia; 6 Machine Learning Applied to Drug Discovery, DrTarget, C/Alejo Carpentier 13, 28806 Alcalá De Henares, Spain; info@doctortarget.com

**Keywords:** drug repurposing, PPAR-α, lipid metabolism, apoptosis, breast cancer, colorectal cancer, broxyquinoline, cefditoren pivoxil, tebipenem pivoxil, artificial intelligence

## Abstract

Drug repurposing offers an efficient strategy to identify novel anticancer agents from marketed compounds. We screened a library of ~1400 approved drugs using a PPARE–PPAR-α luciferase reporter assay to uncover modulators of lipid metabolism that induce cancer cell death without direct binding to PPARα. This primary screen identified 224 compounds inhibiting reporter activity by >50%, of which 26 demonstrated ≥50% cytotoxicity in KAIMRC1 breast cancer and HCT116 colorectal cancer cells at 10 µM after 48 h incubation. AI-based DrTarget analysis classified these hits, with Group 1 comprising four previously unrecognized anticancer agents: antibiotics cefditoren pivoxil, tebipenem pivoxil, broxyquinoline, and cosmetic ingredient 1-hexadecanol. Dose–response studies across KAIMRC1, HCT116, and SW480 cells revealed IC_50_ values as low as ~10 µM, comparable to daunorubicin, a well-known anti-cancer drug. Apoptosis profiling confirmed activation of intrinsic/extrinsic pathways via cytochrome c, SMAC/Diablo, TRAIL-R1/DR4, and phospho-p53 modulation. Based on these findings, we propose the repurposing of Broxyquinoline, 1-Hexadecanol, Cefditoren Pivoxil, and Tebipenem Pivoxil as potential anti-cancer drugs.

## Simple Summary

Cancer cells rely heavily on fat metabolism to survive and grow. By screening over a thousand already-approved drugs, we identified four compounds: an antimicrobial, two antibiotics, and a cosmetic ingredient that can disrupt this fat-based survival mechanism and kill cancer cells. Importantly, none of these drugs were previously known to have anticancer properties. Our findings suggest that medicines already deemed safe for human use could be repurposed as cancer treatments, potentially accelerating the path from laboratory discovery to clinical application.

## 1. Introduction

Discovered in 1990, peroxisome proliferator-activated receptors (PPARs) are transcription factors that belong to the nuclear hormone receptor family. They regulate gene expression once activated by endogenous unsaturated/saturated fatty acids or by synthetic ligands [1]. PPARs heterodimerize with retinoid X receptors (RXRs), resulting in a functional heterodimeric complex. This complex recognizes and directly binds to PPAR response elements (PPREs), located within the promoter regions of target genes [2]. Agonist interaction triggers conformational changes in PPARs, promoting the release of co-repressor proteins and facilitating the recruitment of transcriptional co-activators, ultimately leading to gene activation [3,4]. Similarly, antagonists bind to PPARs’ ligand binding site, restricting binding of endogenous ligands or synthetic agonists that would normally activate these receptors. This antagonist binding also induces conformational change in the PPAR-RXR heteromeric complex, thereby preventing the recruitment of necessary coactivators for transcriptional activation [5].

At present, three PPAR isotypes are known: PPAR-α (also known as NR1C1), PPAR-δ (PPAR-β or NR1C2), and PPAR-γ (NR1C3). Although these isotypes do share a significant degree of homology, they differ in tissue distribution and ligand specificity [3]. There are four major structural components: (1) A/B or ligand-independent domain, including the AF1 region, which is crucial for subtype-specific activity; (2) C or DNA-binding domain (DBD); (3) D or docking cofactors domain; and (4) E or ligand-binding domain (LBD) [6]. Different PPAR isoforms play a crucial role in metabolism-linked pathways, such as glucose, lipid metabolism, and body energy homeostasis [7,8,9]. PPAR-α regulates genes involved in fatty acid uptake, β-oxidation, and ω-oxidation. It regulates apolipoprotein C-III, apolipoprotein A-I, and apolipoprotein A-II [10,11,12]. In the PPAR-α knockout mice model, many genes regulating fatty acid oxidation were found to be downregulated [13].

In recent years, studies have reported that the phenotypes of malignancies such as breast cancer are characterized by altered fatty acid metabolic pathways, including fatty acid transport, de novo synthesis, cholesterol synthesis, and activation [14,15]. Consequently, the involvement of PPAR-α in breast cancer initiation and progression has developed as an area of considerable research interest. PPAR-α antagonists NXT629 [16], MK886 [17], and GW6471 [18], and the agonist fibrates WY-14, 643, and GW7647 [19] are some of the recently developed ligands. Among these, NXT629 has good results in reducing the tumor burden in mouse models for ovarian cancer and melanoma; similarly, GW6471 has shown promising anti-tumor activity under in vitro conditions [16,18].

Drug repurposing has evolved as a smart and efficient strategy to accelerate anticancer drug development, particularly in the context of metabolic vulnerabilities [20]. Existing approved or clinically advanced drugs offer well-characterized pharmacokinetics and safety profiles, thereby reducing the time, cost, and risk associated with de novo drug discovery [21]. In oncology, several successfully repurposed drugs ranging from thalidomide and metformin to non-oncology agents with unanticipated antitumor activity have shown how systematic screening of marketed compounds can uncover new mechanisms of action and expand therapeutic options for patients [22,23]. Building on this concept, exploring the anticancer potential of a pre-existing marketed drug library against PPAR-α-dependent pathways provides a rational framework to identify molecules that perturb lipid metabolism and energy homeostasis in tumor cells, while retaining the translational advantage of drugs that are already in clinical use for other indications. In the present study, we adopted this repurposing-oriented approach to discover previously unrecognized PPAR-α pathway modulators with cytotoxic activity in cancer models.

## 2. Materials and Methods

### 2.1. Chemicals and Reagents

All general biological reagents, including analytical or molecular biology grade buffers and required chemicals, were obtained from Sigma-Aldrich (Milwaukee, WI, USA) and Carl Roth (Karlsruhe, Germany). The approved drug library Tocriscreen 2.0 Max (cat no. 7150) and additional test compounds were procured from Tocris Bioscience (Minneapolis, MN, USA).

### 2.2. Cell Culture

The human breast carcinoma cell line KAIMRC1, established from a 63-year-old Saudi female patient, was previously described [24]. The colorectal cancer cell lines SW620 and HCT116 were obtained from the ATCC, VA, USA. All cell lines were cultured in Dulbecco’s Modified Eagle Medium (DMEM) supplemented with 10% fetal bovine serum (FBS), 50 U/mL penicillin, 50 μg/mL streptomycin, and 2 mM L-glutamine (Gibco, Gaithersburg, MD, USA). Cultures were maintained at 37 °C in a humidified atmosphere containing 5% CO_2_ and 95% relative humidity. Cells were sub-cultured upon reaching 70–80% confluency. Reporter assays were performed using the same culture conditions and FBS concentrations as described above.

### 2.3. Cell Transfection and Luciferase Reporter Assays

KAIMRC1 and HCT116 cells were seeded in 6-well plates at a density of 1 × 10^6^ cells per well in 2 mL of complete medium and allowed to adhere for 2 h. For each transfection, 2.5 μg of PPARE(3)-TK-Luciferase plasmid was used either alone or in combination with 0.5 μg of pcDNA3.1-hPPARα plasmid. The plasmid DNA was diluted in 100 μL of Opti-MEM Medium (Thermo Fisher Scientific, Bartlesville, OK, USA). A total of 0.5 μg of pRL-CMV vector was co-transfected in all samples to normalize for transfection efficiency based on Renilla luciferase activity. Transfection Reagent, FuGENE^®^ 6 (Cat no. E2692; Promega, Madison, WI, USA) was added dropwise (12 μL per reaction) to the DNA mixture, and gently mixed following incubation at room temperature for 8–10 min. The transfection mix was subsequently added to each well containing 1 × 10^6^ cells in 2 mL of fresh culture medium.

Following transfection, cells were incubated overnight at 37 °C in a humidified atmosphere with 5% CO_2_. The next day, the medium was replaced with fresh medium containing either 0% (serum-free) or 10% FBS, as indicated, and incubated for a further 24 h. The next day, cells were transferred to compound plates, and reporter activity was measured using the Dual-Glo^®^ Luciferase Assay System (Promega) following the manufacturer’s instructions.

### 2.4. Cell Proliferation Assays

To obtain the IC_50_, KAIMRC1 and HCT116 cells were treated with a selected panel of commercially available compounds. Briefly, 10,000 cells per well were seeded into 96-well plates and incubated in a 5% CO_2_ incubator overnight. Cells were then treated with test compounds in an 8-point 1:2 serial dilution (100 μM–0.78 μM), followed by 48 h of incubation. CellTitre-GLo^®^ (Promega, Madison, WI, USA) assays were performed after 48 h, and luminescence was measured using the Perkin Elmer Envision Instrument. The luminescence readings were normalized and expressed as a relative percentage. The data was analysed, and the half-maximal inhibitory concentration (IC_50_) was determined using custom Python scripts (using 3PL, 4P models). Error bars denote standard deviation (SD).

### 2.5. Human Apoptosis Array

KAIMRC1 cells were seeded in 6-well plates and treated for 48 h with either vehicle control (DMSO), Broxyquinoline, 1-Hexadecanol, Cefditoren pivoxil, Tebipenem pivoxil, and Daunorubicin as a positive control,10 μM each. Following treatment, cell lysates were collected for analysis. Apoptosis-related protein expression profiling was performed using the Human Apoptosis Array Kit (Cat. #ARY009, R&D Systems, Minneapolis, MN, USA) to identify apoptosis-related proteins potentially associated with PPAR-α inhibition. Each membrane within the array contained 22 distinct capture antibodies spotted in duplicate. All procedures were conducted according to the manufacturer’s protocols. In brief, after blocking for 1 h, each membrane was incubated overnight at 4 °C with 400 μg of cell lysate of treated cells. Following incubation, membranes were washed 3 times with wash buffer and incubated with detection antibody cocktail for 1 h. After washing, each membrane was treated with streptavidin-HRP for 30 min and was developed using Chemi reagent. The BIORAD ChemiDoc MP imaging (Hercules, CA, USA) system was used to take the images of the blots. For image analysis, ImageJ/Fiji was employed to read the dots’ intensity in terms of Integrated Density [25]. Custom-made Python scripts were used to analyse and plot the raw data.

### 2.6. Molecular Docking Studies

The crystal structure of the PPAR-α ligand-binding domain (LBD) co-crystallized with the antagonist GW6471 (PDB ID: 1KKQ, resolution 3 Å) was obtained from the Protein Data Bank [26,27]. The structure contains four LBD copies corresponding to chains A–D. Chain C, which includes the co-repressor peptide (silencing mediator for retinoid and thyroid hormone receptors [SMRT]) and the co-crystallized antagonist, was selected for molecular docking based on previous structural studies [27]. Protein preparation was performed using Schrödinger’s Protein Preparation Workflow [28]. Bond orders were assigned using the Chemical Component Dictionary (CCD), termini were capped, and missing side chains and loops were completed using Prime. Protonation states were generated at pH 7 ± 2 using Epik, and hydrogen bond networks were optimized using PROPKA with water sampling and hydrogen minimization. The protein structure was energy-minimized using the Optimized Potentials for Liquid Simulations 4 (OPLS4) force field, and water molecules positioned beyond 5 Å from the anticipated ligand binding site were removed.

Three-dimensional structures of the test compounds, broxyquinoline (PubChem CID: 2453), 1-hexadecanol (CID: 2682), cefditoren pivoxil (CID: 6437877), and tebipenem pivoxil (CID: 9892071), were retrieved from the PubChem database. All ligands and the co-crystallized antagonist GW6471 were prepared using Schrödinger’s LigPrep module. The OPLS4 force field was applied, and ligand protonation states at pH 7 ± 1 were generated using Epik. Chirality was determined from the three-dimensional structures.

The receptor grid was generated using the co-crystallized antagonist as the spatial reference, with a van der Waals radius scaling factor of 1.0 and a partial charge cutoff of 0.25. Molecular docking was performed using Schrödinger’s Glide tool employing the extra precision (XP) method. Sampling of nitrogen inversions and ring conformations was enabled, and Epik state penalties were applied to docking scores. Post-docking minimization was applied, generating three poses per ligand. All poses were visually evaluated for correct orientation within the binding pocket and ranked based on docking scores and interactions with key residues.

### 2.7. TR-FRET-Based Competitive Receptor Binding Assay

A TR-FRET-based competitive receptor binding assay was performed to determine ligand binding to PPAR-α using the Thermo Scientific LanthaScreen™ Competitive Binding Assay Kit (Catalogue no. PV4892). The binding affinities of four test compounds, i.e., Broxyquinoline, 1-Hexadecanol, Cefditoren pivoxil, and Tebipenem pivoxil, were assessed. Moreover, GW6471 and GW7647 were used as a PPAR-α antagonist and agonist (positive control), respectively, and DMSO as a negative control. Briefly, 20 μL of a 2× solution of each test compound and control compound was added to 96-well plates, followed by 10 μL of 4× Fluormone™ Pan-PPAR Green and 10 μL of 4× PPAR-α-LBD/Tb-anti-GST antibody. Plates were gently mixed on an orbital shaker for 30 s, covered, and incubated at room temperature for 1 h. The fluorescence emission-absorbance was measured using SpectraMax Instruments (Molecular Devices, San Jose, CA, USA). Two wavelengths per well were taken with the instrument setting as follows: 340 nm excitation filter, 30 nm bandwidth, 520 nm emission filter, 25 nm bandwidth, 495 nm emission filter, 10 nm bandwidth; delay time of 100 µs, an integration time of 200 µs. The results were expressed as a ratio by dividing the emission signal at 520 nm by the emission signal at 495 nm.

### 2.8. Bioinformatics Analysis Using DrTarget Platform

Target identification and off-target profiling were conducted using the DrTarget machine learning platform (https://doctortarget.com/solutions, accessed on 18 June 2026). This tool uniquely integrates multiple data sources and comprehensively characterizes test compounds and target interactions. The main key features that make it a valuable tool include ChEMBL activity scoring function, assay classification and phenotyping, Mechanism of Action (MoA) Mapping, clinical trial status, and comprehensive ChEMBL database search.

DrTarget starts with identifying all ChEMBL identifiers associated with submitted test compound names to retrieve associated bioactivity data independent of structural representation. Moreover, it also provides normalized activity scores for around 20 × 10^6^ compounds, substantially more than ChEMBL’s native (~3 × 10^6^) pChEMBL values. It standardizes heterogeneous potency measurements like EC_50_, IC_50_, and percent inhibition to a comparable scale. For assay classification and phenotyping characterization, all ChEMBL assays (>95%) were curated into a three-level disease classification system. For all the cancer-related phenotypes, classification includes cell death/growth, cancer type, and specific cell line or tissue. This annotation enabled the identification of disease-relevant bioactivity. For Mechanism of Action (MoA) Mapping, canonical mechanisms of action were assigned to each compound. This includes target pair using Open Targets data, incorporating information on pharmacological targets, mode of action, and drug modality. This module detected whether compounds have established MoAs and also predicted potential clinical relevance. The platform also determined whether each test molecule has been evaluated in clinical trials. Furthermore, it also identified associated disease indications using Open Targets clinical trial data, providing a translational context for experimental compounds. It also performed a comprehensive ChEMBL database search. All ChEMBL interactions encompassing in vitro and in vivo experimental records were queried. The resulting database combines Activities, Assays, Cell Dictionary, and Molecule Dictionary tables with UniProt mapping, along with DrTarget scoring and classification annotations, enabling identification of off-target binding and potential safety liabilities.

## 3. Results and Discussion

### 3.1. Constitutive Activation of the PPARα Pathway in KAIMRC1 and HCT116 Cell Lines

**Figure 1:**
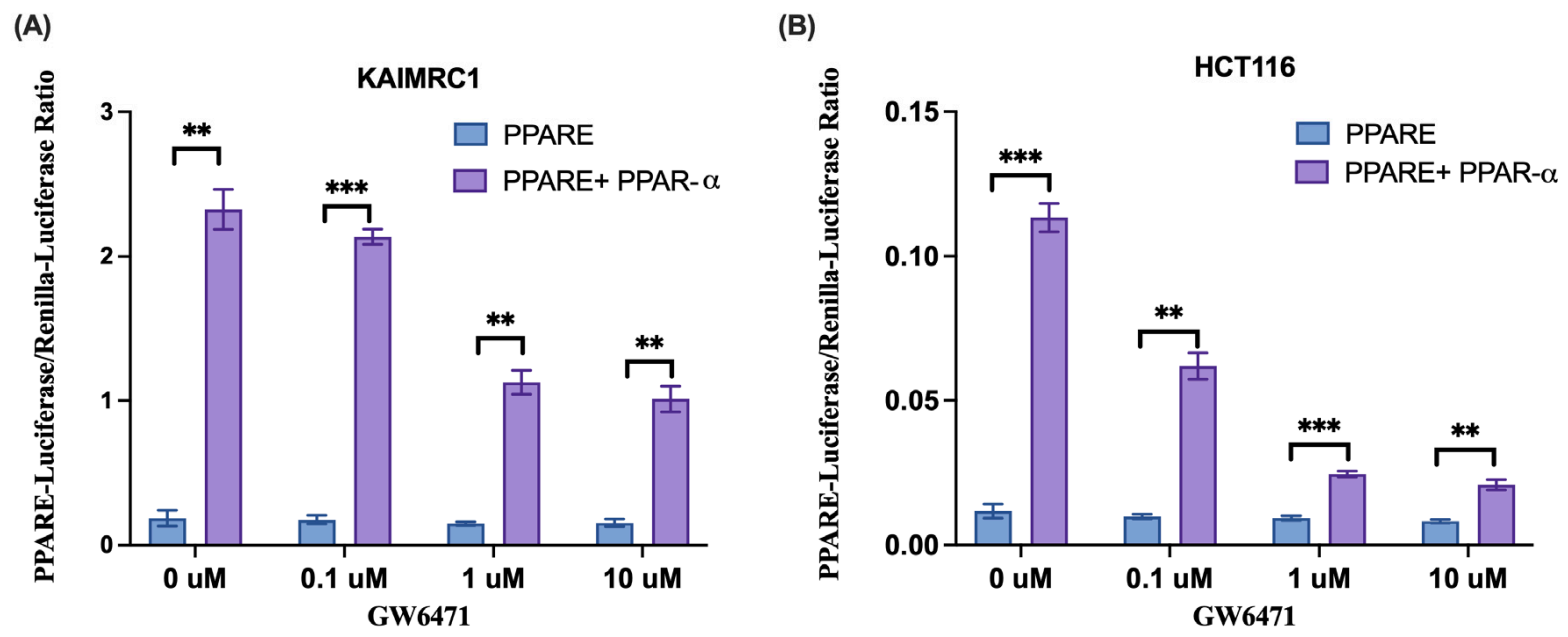
The ratio of PPARE-Luciferase/Renilla-Luciferase in (**A**) KAIMRC1 and (**B**) HCT116 is plotted. Breast cancer KAIMRC 1 and colon cancer HCT116 Cells were transfected with PPARE-Luciferase (PPARE) alone or with PPAR-α (PRA). Later, they were treated with PPAR-α antagonist (GW6471). Bars represent mean ± SD (*n* = 3, Welch’s two-tailed *t*-test. *** *p* < 0.001, ** *p* < 0.01, vs. PPARE control.).

In our earlier work, we characterized the activity of nuclear receptors in the KAIMRC1 breast cancer cell line and found distinct activation patterns compared with MCF7 and MDA-MB-231 cells [24]. These observations signified the importance of KAIMRC1 as a novel breast cancer model, complementing existing cell lines. In another study, we further assessed PPAR-α and PPAR-γ activity and found that the PPAR signaling pathway, particularly PPAR-α, is constitutively active in KAIMRC1 cells [19]. In this present work, we also extended this particular analysis to include the HCT116 cell line. As shown in Figure 1, co-transfection with PPAR-α receptors markedly enhanced PPARE-Luciferase reporter activity. Consistent with previous reports, treatment with the PPAR-α antagonist GW6471 significantly reduced reporter activity in a concentration-dependent manner in both KAIMRC1 and HCT116 cells. These findings support the previous notion that cancer cells activate PPAR-α-driven pathways to enhance fatty acid metabolism as rapidly proliferating cancerous cells require it for energy during metabolic stress, thereby providing an auxiliary energy source for survival [29]. This concept underpins our subsequent screening strategy, in which we used PPARE-Luciferase activity as a functional readout to discover novel modulators of the PPAR-α pathway with cytotoxic activity in KAIMRC1 and other cancer cells.

### 3.2. Identification of Inhibitors Targeting the PPAR-α Signaling Pathway in KAIMRC1 Cells

We exploited this cancer cell survival mechanism to screen for drug molecules capable of suppressing PPARE-Luciferase reporter activity in KAIMRC1 cells following PPAR-α activation. The underlying rationale was that compounds inhibiting reporter activity could also induce cytotoxicity in KAIMRC1 cells by affecting fatty acid synthesis, thereby serving as potential lead drug candidates. Our intention was not to discover classical ligand-binding PPAR-α antagonists, but rather to identify functional inhibitors of the PPAR-α signalling axis, similar to recent efforts to find nuclear receptor modulators that act through non-canonical mechanisms [19,30,31]. A marketed drug library comprising 1413 compounds was screened at a final concentration of 10 µM for 24 h. The reporter activity data identified 224 compounds that reduced PPARE-Luciferase activity by more than 50% without significantly affecting Renilla Luciferase, which served as an internal normalization control.

Numerous studies have reported that PPAR-α exerts pleiotropic and context-dependent roles in cancer. Pharmacologic or genetic inhibition of PPAR-α can trigger G_0_/G_1_ arrest, apoptosis, and enhanced sensitivity to metabolic stress in renal cell carcinoma and other cancer models [32,33,34]. Moreover, synthetic PPAR agonists and fibrates show receptor-independent mitochondrial effects that reshape cancer cell bioenergetics, further emphasizing that PPAR pathway modulation may extend beyond simple ligand–receptor binding [35]. In this context, compounds that inhibit PPARE–luciferase activity in KAIMRC1 cells may be acting at multiple levels. The relatively high hit rate in our primary screen (224 of 1413 approved drugs) is supportive of the view that nuclear receptors, including PPARs, could be modulated as off-target by existing therapeutics. Similar luciferase-based reporter assays have been previously used for high-throughput discovery of ligands for PPAR-γ and PPAR-β/δ in metabolic disease and steatosis models [36]. Our data extend this strategy in an oncology-relevant context by using a breast cancer line in which PPAR-α is endogenously coupled to lipid metabolism and survival. The fact that all of these molecules are marketed drugs provides an immediate translational advantage. The pharmacokinetics, safety, and in some cases, central nervous system penetration is already defined.

### 3.3. Inhibition of the PPAR-α Pathway Leads to Cancer Cell Death

The shortlisted drug candidates were further evaluated for their ability to induce cancer cell death under in vitro conditions. Here, compounds that reduced PPARE-Luciferase activity by more than 50% were tested for their anticancer potential. KAIMRC1 and HCT116 cells were treated with selected drugs at a concentration of 10 µM for 48 h, and cell viability was quantified using the CellTiter-Glo luminescent assay. It reports metabolically activate cells in terms of total ATP. In this assay, 26 compounds were able to reduce the viability of more than 50% of either KAIMRC1 or HCT116 cells. These 26 compounds were then prioritized as promising lead candidates for further investigation (Table 1).

This reduction in the number represents a substantial enrichment relative to the primary reporter screen. Here, only a subset of PPARE-suppressing drugs was effective in inducing cytotoxicity at pharmacological concentrations. Several compounds displayed preferential activity in KAIMRC1 versus HCT116 cells (and vice versa), suggesting that dependence on PPAR-α-driven lipid metabolism and downstream survival programs is context-specific and influenced by the underlying genomic background and tissue origin. Conversely, PPARE-low “hits” that failed to impair viability likely act upstream or in parallel to PPAR-α without critically compromising the core metabolic circuitry required for short-term survival. Together, these findings support a working model in which functional inhibition of the PPAR-α axis is necessary but insufficient for robust cancer cell killing.

### 3.4. AI-Guided Oncological Annotation and Compound Classification Using the DrTarget Platform

In order to further triage the 26 cytotoxic hits rationally, all compounds were annotated using the DrTarget machine learning platform. This AI tool integrates bioassay data, pharmacological activity records, mechanistic annotation, and clinical indication information from multiple curated databases (see Section 2). The platform assigned each compound to one of three groups on the basis of its oncological history (Table 1). Compounds in the Group 1 category have no prior link to cancer therapy or cancer clinical trials. Group 2 compounds have no registered clinical trials in oncology but have published evidence of cancer cell killing. Compounds with established or ongoing oncological use or clinical evaluation are in group 3. This grouping approach enabled us to focus downstream mechanistic investigation on the most genuinely new anticancer candidates and to keep the compounds whose anticancer activity had already been reported.

Group 1 compounds were selected as the primary focus for all downstream investigations for two reasons. First, they represent genuinely novel anticancer candidates, unlike Group 2 and Group 3 compounds, whose cytotoxic activity has already been documented to varying degrees in the literature. On the other hand, Group 1 compounds have no reported oncological association, meaning that any mechanistic findings from this study would constitute an original contribution. Second, pursuing compounds in Groups 2 and 3 would largely recapitulate existing cancer biology rather than expand it, reducing the translational value of the work. Four compounds, namely cefditoren pivoxil, tebipenem pivoxil, 1-hexadecanol, and broxyquinoline, assigned to Group 1, represent the highest-priority repurposing candidates. Cefditoren pivoxil, which is an orally administered third-generation cephalosporin antibiotic approved for the treatment of community-acquired pneumonia and skin infections [37]. Tebipenem pivoxil is an oral carbapenem developed for multidrug-resistant bacterial infections [38]. 1-hexadecanol (cetyl alcohol) is a long-chain saturated fatty alcohol used primarily as an emollient and emulsifier in pharmaceutical and cosmetic formulations and has not been investigated as an anticancer drug in human studies. Broxyquinoline is a halogenated hydroxyquinoline derivative used for its antimicrobial properties [39]. The absence of any prior oncological association for all four molecules underscores their novelty as anticancer candidates and validates the utility of AI-guided curation for identifying unconventional repurposing opportunities.

None of the four Group 1 compounds displaced the fluorescent tracer from the PPARα ligand-binding domain in the TR-FRET assay. Also, their docking scores along with binding-pocket interactions were markedly inferior to those of the reference antagonist GW6471. Suggesting direct PPARα antagonism, an unlikely mechanism here. Instead, these compounds may reduce PPARE–luciferase reporter activity indirectly by perturbing the supply of endogenous lipid ligands that sustain constitutive PPARα activation in KAIMRC1 cells (Figure 1) [19]. To explore this, DrTarget pathway analysis was further applied to all four compounds by linking their ChEMBL-derived protein-target interactions to Reactome pathway annotations (see Supplementary Table S1). This analysis identified multiple lipid metabolic routes as predicted targets, including arachidonate metabolism, prostaglandin and thromboxane synthesis, DHA-derived specialized pro-resolving mediator (SPM) biosynthesis, and broader fatty-acid metabolic pathways. These pathways together generate several established endogenous PPARα ligands like arachidonic acid and its eicosanoid derivatives [40], as well as prostaglandins and thromboxanes [41]. Attenuation of which would be expected to reduce receptor-driven transcription and suppress PPARE–luciferase activity in the absence of exogenous agonist treatment.

**Figure 2:**
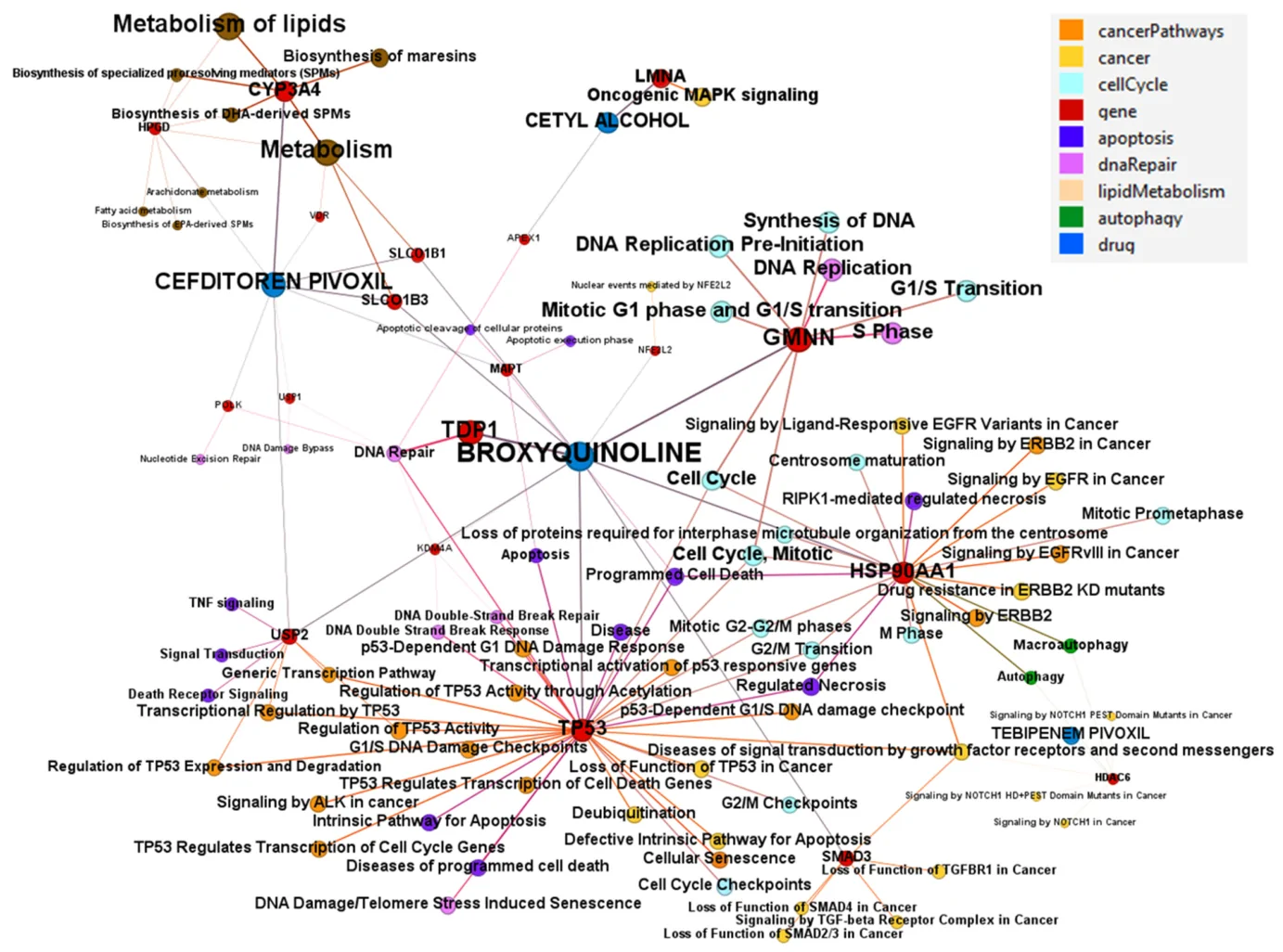
Network graph showing the molecule–target interactions recorded in ChEMBL. Node size and edge width are based on ChEMBL scores aggregation as described above. For the target-molecule pairs (edges), the interaction score is a weighted value based on the average and max ChEMBL score, plus the number of ChEMBL assays for each pair. Node sizes are represented according to interaction scores aggregated to the molecule, pathway, and protein target levels. Node colors are based on DrTarget pathways classification as described in the legend.

DrTarget pathway analysis also highlighted involvement of canonical cell-death programs. As illustrated in Figure 2 and summarized in Supplementary Table S1, the four compounds collectively modulate pathways encompassing caspase-mediated cleavage of cytoskeletal proteins, apoptotic cleavage of cellular targets, TNF receptor signaling, BH3-domain activation, autophagy induction, and p53-related tumor-suppressor signaling. Given that lipids are a critical energy source for rapidly proliferating cancer cells such as KAIMRC1, their bioenergetic dependence means that inhibition of fatty-acid synthesis and oxidation pathways is itself cytotoxic [42,43]. Taken together, the DrTarget analysis supports a multi-mechanism model in which these Group 1 compounds converge on KAIMRC1 cell death through at least three interconnected axes: (i) indirect suppression of PPARα-driven transcription via depletion of endogenous lipid ligands; (ii) disruption of lipid-based energy provision; and (iii) direct or downstream activation of pro-apoptotic and autophagic signaling cascades. This network-level picture informed our subsequent selection of apoptosis-profiling experiments to validate computational predictions experimentally.

### 3.5. IC_50_ Determination for Compound-Induced Cell Death

**Figure 3:**
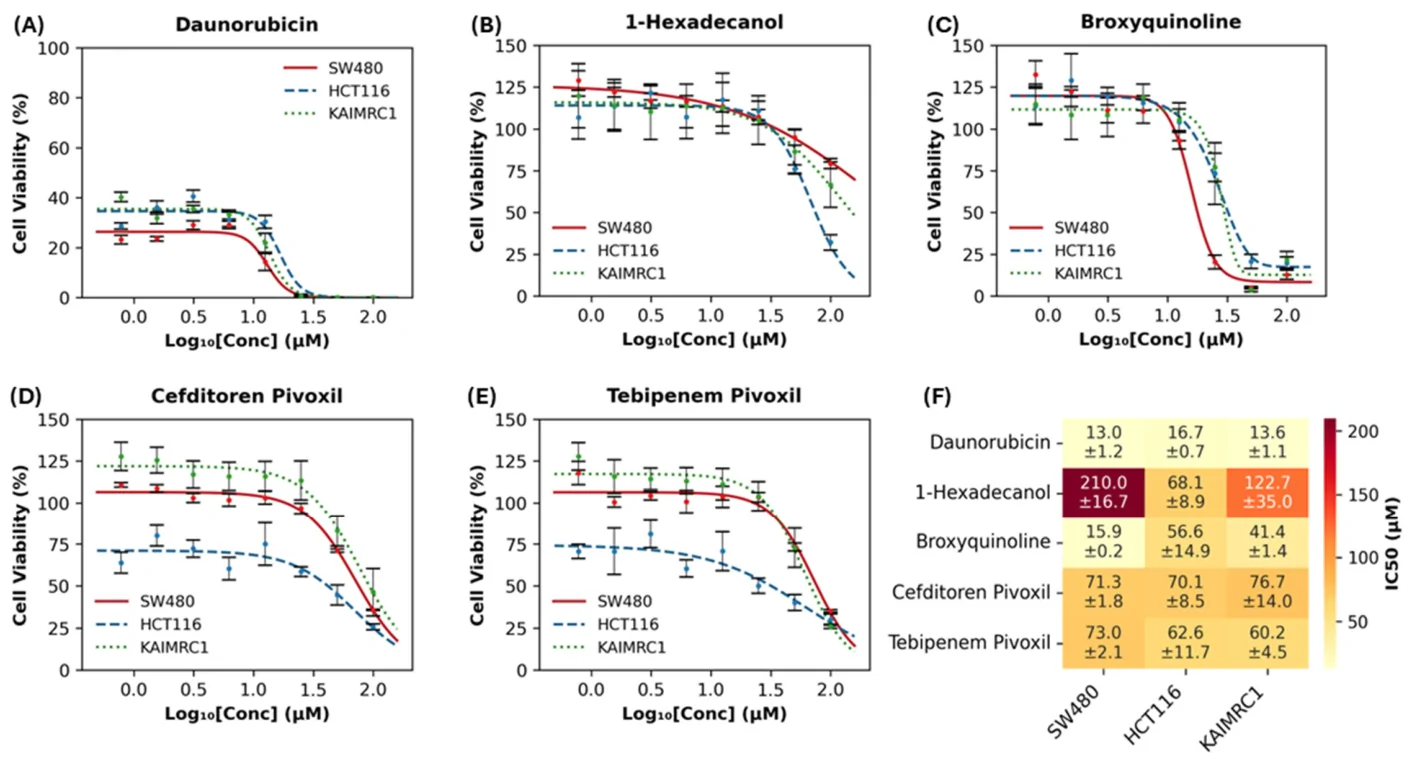
Curve plots of (**A**) positive and (**B**–**E**) selected compounds of Group 1 (No past or ongoing clinical trials in cancer and not known in the literature to kill cancer cells), were tested in killing SW480, HCT116, and KAIMRC1 cells in a dose-dependent manner. The cell killing was measured using CellTitre-Glo after 48 h of treatment as indicated. (**F**) Heat plot summarising IC50 (µM) ± SEM values of group 1 compounds. Data points represent the mean of three independent biological replicates normalised to DMSO vehicle control; error bars denote the standard error of the mean (SEM, *n* = 3). Dose–response curves were fitted to logistic models (3PL/4PL/5PL) using non-linear least squares regression, with the best-fit model selected by the Akaike Information Criterion (AIC). IC_50_ values in panel F are displayed as mean ± SEM calculated from replicate curve fits.

To quantify the cytotoxic potential of our lead set, we performed dose–response analyses in three PPAR-relevant epithelial cancer models: the colorectal lines SW480 and HCT116, along with the breast cancer-derived KAIMRC1 line, all of which express functional PPAR signalling. Cells were exposed to an eight-point, 1:2 serial dilution series (maximum concentration 100 µM) of each compound for 48 h, and viability was measured using the CellTiter-Glo luminescent ATP assay, a sensitive approach to quantify the number of metabolically active cells. Concentration–response curves were fitted to derive half-maximal inhibitory concentrations (IC_50_), and the resulting values are summarised as both sigmoidal killing plots and a heat-map representation (Figure 3).

The Figure 3 panel comprised killing curves of positive control, daunorubicin, together with four Group 1 candidates: broxyquinoline, 1-hexadecanol, cefditoren pivoxil, and tebipenem pivoxil, that have no previously reported antitumour activity or registered cancer trials and have not, to our knowledge, been previously described as cytotoxic agents in solid-tumour models. As expected, daunorubicin produced steep killing curves across all three cell lines, with IC_50_ values in the low-micromolar range (≈13–17 µM), which also validates the assay conditions (Figure 3A,F).

Interestingly, despite their current use only as antimicrobials and excipient-like lipids, all four Group 1 compounds were able to reduce cell viability, with IC_50_ values spanning approximately 16–210 µM depending on the cell line (Figure 3B–F). Two orally bioavailable β-lactam antibiotics, Cefditoren pivoxil (Figure 3D,F) and tebipenem pivoxil (Figure 3E,F), showed reproducible, moderate cytotoxicity (ranging around 60–76 µM) in SW480, HCT116, and KAIMRC1 cells, which is notable given the emerging appreciation that certain antibiotics can be repurposed as metabolic or cell-cycle modulators in cancer [44,45,46]. Similarly, the saturated long-chain fatty alcohol, 1-hexadecanol, also decreased viability at relatively high concentrations varying from 68 to 210 µM (Figure 3B,F). These results are also consistent with the growing evidence that disturbed lipid handling and membrane composition can trigger stress responses and apoptosis in tumour cells. This process is tightly intertwined with PPAR-regulated lipid metabolism [47].

Among the repurposing candidates, broxyquinoline, a halogenated quinoline derivative, displayed the most noticeable antitumour activity, with IC_50_ values of ~16–56 µM (Figure 3C,F). Quinoline scaffolds are increasingly recognised as privileged structures in oncology. Multiple analogues are reported to interfere with DNA topology, cell-cycle regulators, and mitochondrial function in diverse cancer types, yet broxyquinoline itself has not previously been linked to direct anticancer activity [48,49]. Its relatively low IC_50_ values compared with the other Group 1 molecules, coupled with the breadth of activity across colon and breast models, position broxyquinoline as a particularly compelling starting point for mechanistic dissection. Especially in the context of PPAR-dependent metabolic rewiring and mitochondrial vulnerabilities. Overall, these data indicate that several clinically used, non-oncologic drugs can elicit measurable cytotoxicity in PPAR-competent cancer cells.

### 3.6. TR-FRET Competitive Binding Confirms Indirect PPAR-α Modulation by Group 1 Compounds

**Figure 4:**
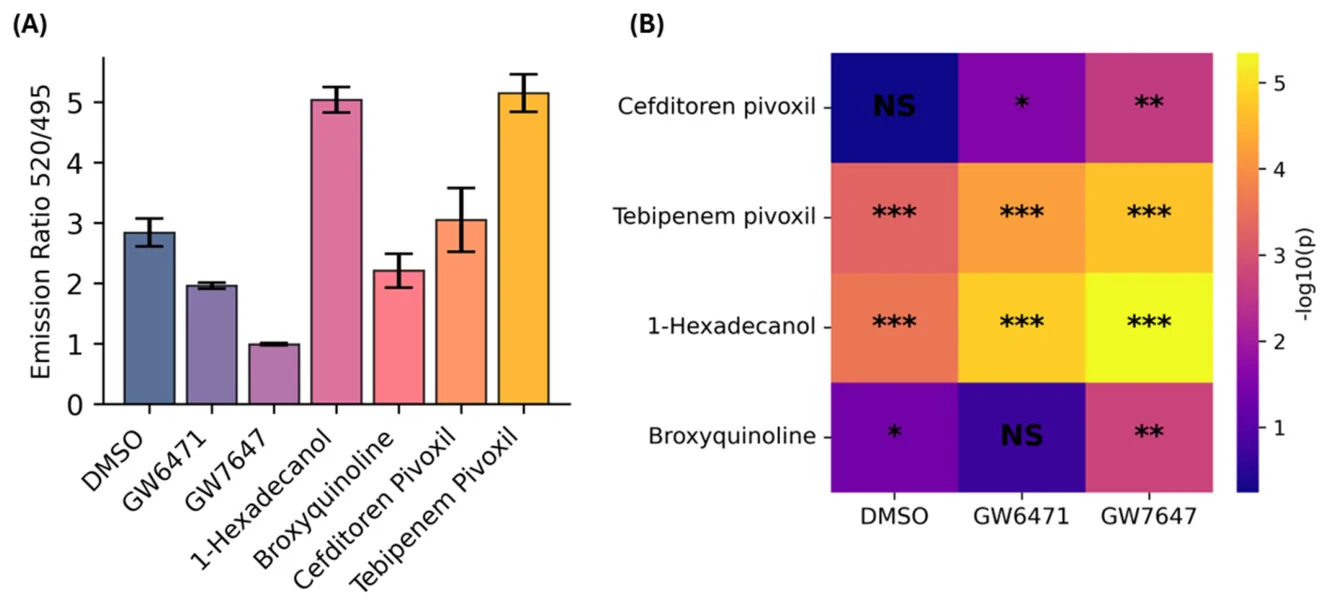
(**A**) Mean 520/495 emission ratios after 1 h incubation with DMSO, the PPAR-α antagonist GW6471, the PPAR-α agonist GW7647, and the repurposed compounds cefditoren pivoxil, tebipenem pivoxil, 1-hexadecanol, and broxyquinoline at 10 µM. Bars represent the mean ± SD. (**B**) Heatmap showing the statistical significance of Group 1 compounds relative to DMSO, GW6471, and GW7647 based on pairwise *t*-tests of the 520/495 ratios. Stars indicate significance levels (* *p* < 0.05, ** *p* < 0.01, *** *p* < 0.001), and NS indicates not significant.

To assess whether the candidates interact directly or indirectly with the ligand-binding domain (LBD) of PPAR-α, we employed a TR-FRET competitive binding assay. Here, binding of the fluorescent tracer to PPAR-α generates a high 520/495 nm emission ratio, and displacement of the tracer by a competing ligand leads to a reduction in this ratio [50]. As expected, the control agonist GW7647 and the antagonist GW6471 both markedly decreased the 520/495 signal relative to the DMSO control at 10 µM, confirming robust, direct engagement of the LBD and validating the dynamic range of the assay (Figure 4).

In contrast, as shown in Figure 4, the Group 1 compounds 1-hexadecanol, broxyquinoline, cefditoren pivoxil, and tebipenem pivoxil did not follow this tracer-displacement profile. Cefditoren pivoxil yielded a TR-FRET ratio similar to DMSO, indicating no meaningful competition for the ligand-binding pocket. Tebipenem pivoxil and 1-hexadecanol produced a significant increase in the 520/495 ratio, a pattern inconsistent with competitive tracer displacement and more likely attributable to compound-related optical interference or non-specific stabilisation of the assay complex at 10 µM. Broxyquinoline caused only a small reduction in the ratio that, although antagonist-like in magnitude, was markedly weaker than that of GW7647 or GW6471. Overall, these patterns are more compatible with a lack of high-affinity competition for the canonical ligand-binding pocket and/or weak assay interference than with classical PPAR-α ligands. When integrated with the PPARE-luciferase and viability data, the TR-FRET results therefore support a model in which the Group 1 drugs modulate PPAR-α–dependent transcription and cancer cell survival predominantly through indirect mechanisms, potentially via upstream metabolic pathways, mitochondrial function, or co-regulator networks rather than through strong, direct occupation of the nuclear receptor LBD.

### 3.7. Molecular Docking of Group 1 Compounds with PPAR-α

To date, there is only one X-ray crystal structure of the PPAR-α LBD that is co-crystallized with an antagonist in an inactive state (PDB ID: 1KKQ) [27]. The structure is a complex between the PPAR-α receptor, the antagonist GW6471, and the SMRT co-repressor peptide. In previous studies, agonists form an essential hydrogen bond with Y464 on the activation helix 12 (H12) [51]. On the other hand, the antagonist GW6471 lacks this interaction and instead pushes H12 out, rendering the receptor in an inactive conformation [27]. Moreover, the antagonist-induced LBD conformation was found to be favorable for the binding of the SMRT co-repressor peptide to the receptor, further contributing to receptor inactivation [27]. In the binding site, GW6471 was also found to wrap around C276 of H3 in a U-shaped conformation [27]. These findings suggest that for a molecule to function as a PPARα antagonist, it must adopt an appropriate orientation to push H12 out, thereby preventing receptor activation. Moreover, GW6471 and other antagonists form hydrogen bonds with Y314, which has also been linked to antagonist activity [27,52].

**Figure 5:**
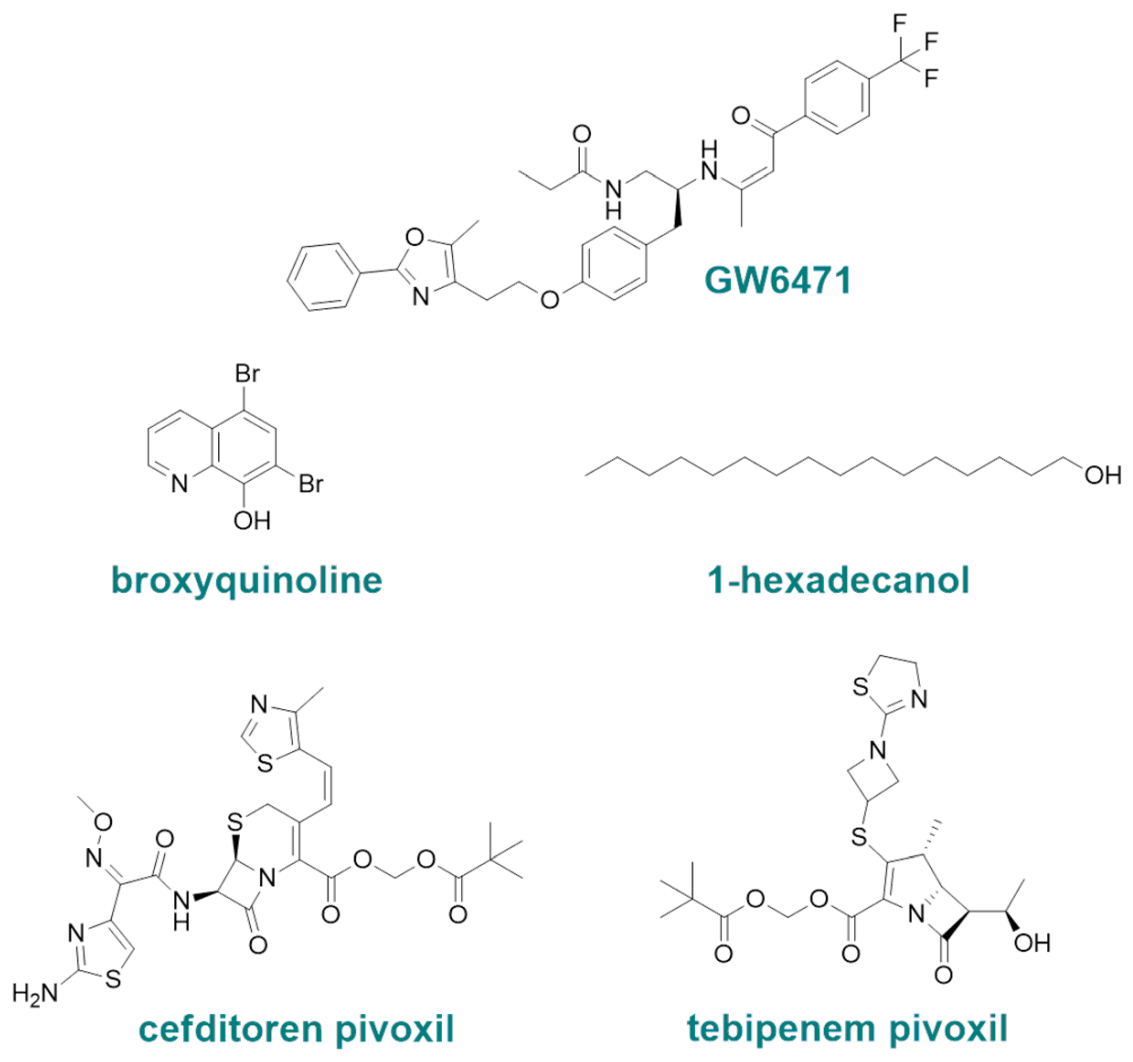
Structures of GW6471 and the four group 1 compounds.

**Figure 6:**
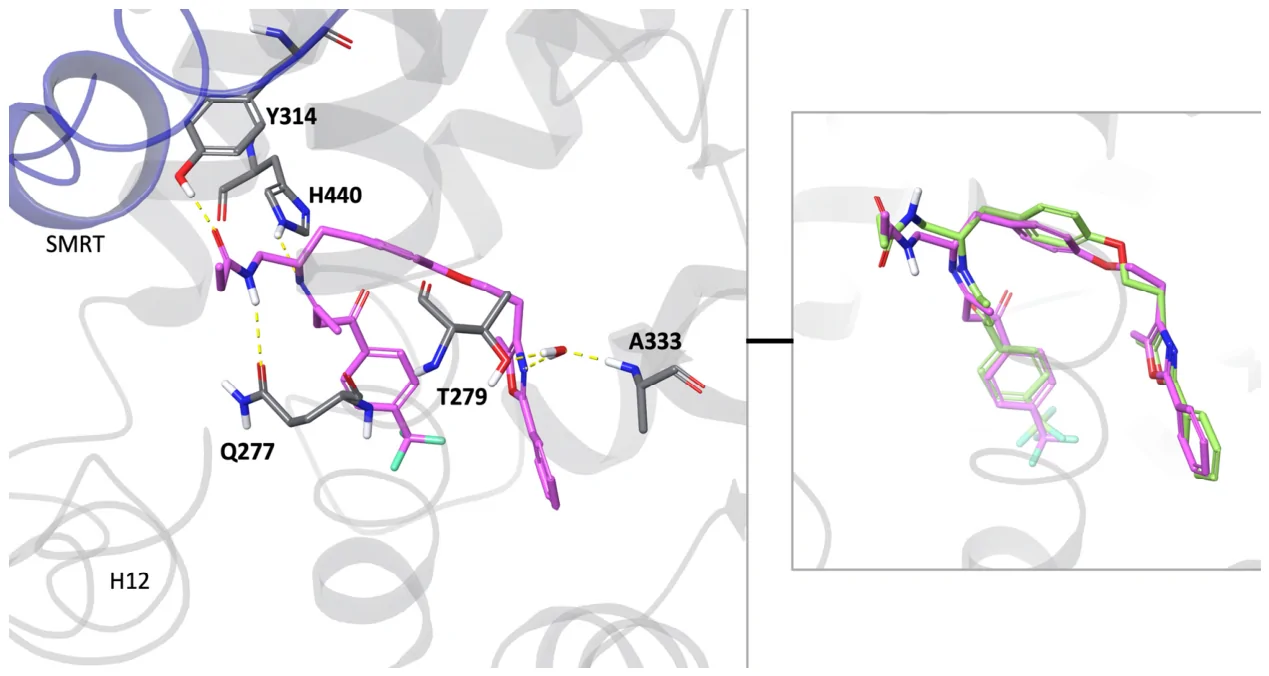
The docking pose of GW6471 (magenta sticks) in the LBD of PPARα (grey cartoon, interacting residues are shown as grey sticks). The SMRT co-repressor is represented as a blue cartoon, and hydrogen bonds are shown as yellow dashed lines. The inset shows the overlay of the X-ray crystal structure pose (lime sticks) and the docking pose.

To validate the molecular docking protocol, the co-crystallized antagonist GW6471 (Figure 5) was docked into the X-ray crystal structure of PPAR-α (PDB ID: 1KKQ). The docked pose for GW6471 adopted a U-shaped conformation and had a root mean square deviation (RMSD) value of 0.81, indicating good reproducibility of the X-ray crystal structure binding mode (Figure 6). The docking score of GW6471 was −13.95 kcal mol^−1^, and it formed hydrogen bonds with Y314, H440, and Q277, and water bridges with A333 and T279.

**Figure 7:**
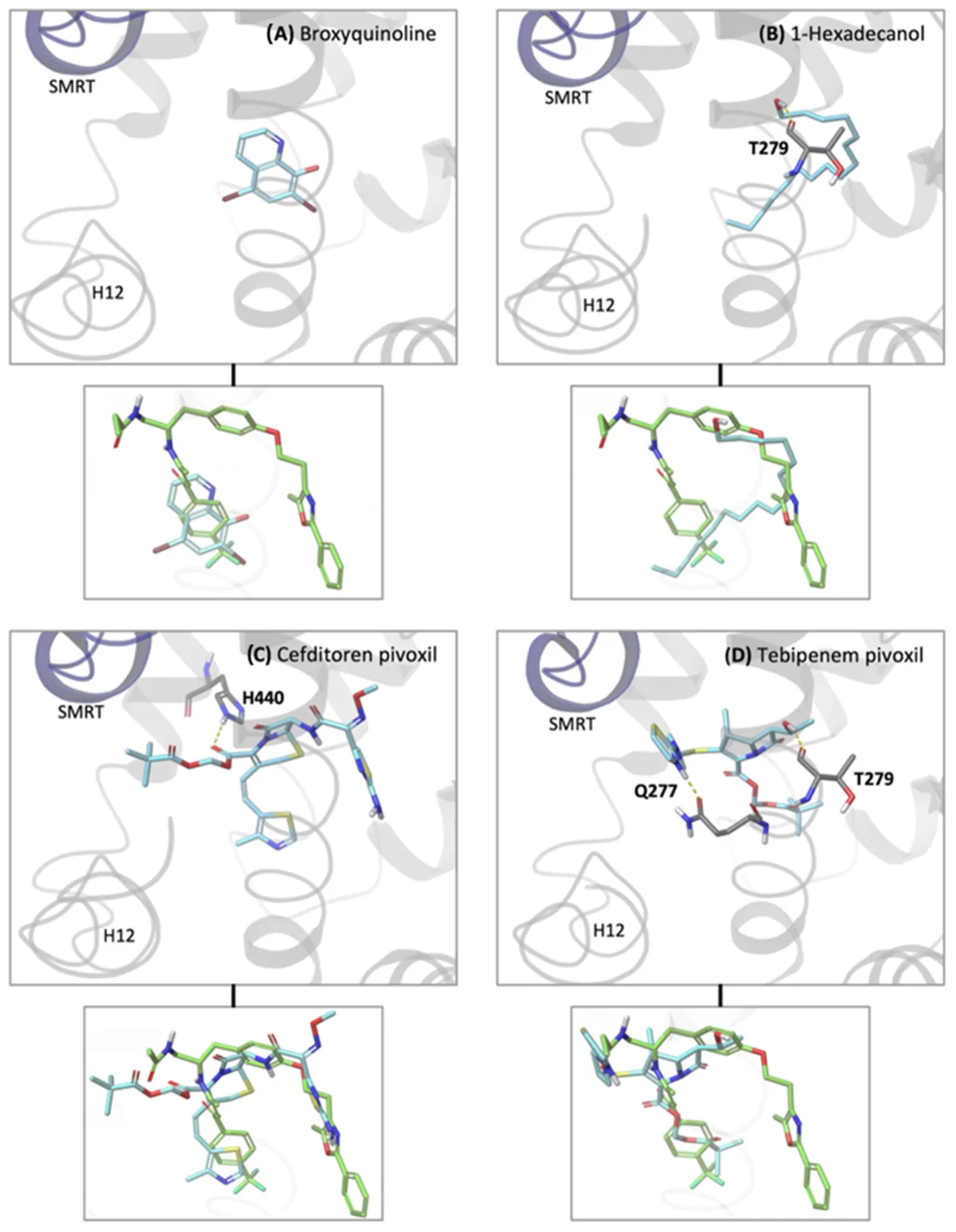
The docked poses of (**A**) broxyquinoline, (**B**) 1-hexadecanol, (**C**) cefditoren pivoxil, and (**D**) tebipenem pivoxil (cyan sticks). Overlays for each of these compounds with the X-ray crystal structure pose of GW6471 (lime sticks) are shown in the insets. The LBD of PPARα is represented as a grey cartoon, interacting residues as grey sticks, the SMRT co-repressor as a blue cartoon, and hydrogen bonds as yellow dashed lines.

To investigate whether broxyquinoline, 1-hexadecanol, cefditoren pivoxil, and tebipenem pivoxil (Figure 5) could function as antagonists of PPAR-α, they were docked into its LBD of PPAR-α (Figure 7). Broxyquinoline had a docking score of −5.87 kcal mol^−1^, and it did not form any interactions with any residues (Figure 7). 1-Hexadecanol had a docking score of −4.16 kcal mol^−1^ and formed a hydrogen bond with T279 (Figure 7). Interestingly, both broxyquinoline and 1-hexadecanol were bound in a region of the binding pocket that does not allow interaction with Y314 nor displacement of H12.

Cefditoren pivoxil had a docking score of −7.73 kcal mol^−1^ and formed a hydrogen bond with H440, adopting a U-shaped conformation similar to that of the antagonist GW6471 (Figure 7C). Tebipenem pivoxil had a docking score of −6.19 kcal mol^−1^ and formed hydrogen bonds with Q277 and T279 while adopting a T-shaped conformation (Figure 7D). Interestingly, the structures of both cefditoren pivoxil and tebipenem pivoxil extended into the region of the binding site that supports pushing H12 out; however, neither compound formed the Y314 hydrogen bond commonly observed for PPAR-α antagonists. The lack of this important interaction renders cefditoren pivoxil and tebipenem pivoxil unlikely to function as PPARα antagonists.

Notably, the co-crystallized antagonist GW6471 had a much more favourable docking score compared to all four compounds, supporting its function as an antagonist and suggesting weak predicted binding affinities of the four group 1 compounds. Overall, the unfavorable docking scores and the lack of interactions with Y314 suggest that broxyquinoline, 1-hexadecanol, cefditoren pivoxil, and tebipenem pivoxil are unlikely to act as antagonists of the PPAR-α receptor. Broxyquinoline and 1-hexadecanol also do not bind in a way that could displace H12, in addition to having the weakest predicted binding affinities based on their docking scores.

### 3.8. Effect of PPAR-α Pathway Inhibitors on Apoptosis Pathways

To further understand the mechanism of cancer cell death, we further examined activation of apoptosis pathways in KAIMRC1 cells following treatment with the four shortlisted drugs. Antibody array dot blot profiling of apoptosis-related proteins uncovered that several canonical components of the death receptor, along with mitochondrial apoptosis pathways, were induced by the drug treatments (Supplementary Figure S1). Among the p53 axis, phosphorylation of p-p53 at S15 and S392 increased significantly in response to cefditoren pivoxil and tebipenem pivoxil, with intermediate induction by daunorubicin and hexadecanol, while DMSO and broxyquinoline elicited only modest signals [53].

**Figure 8:**
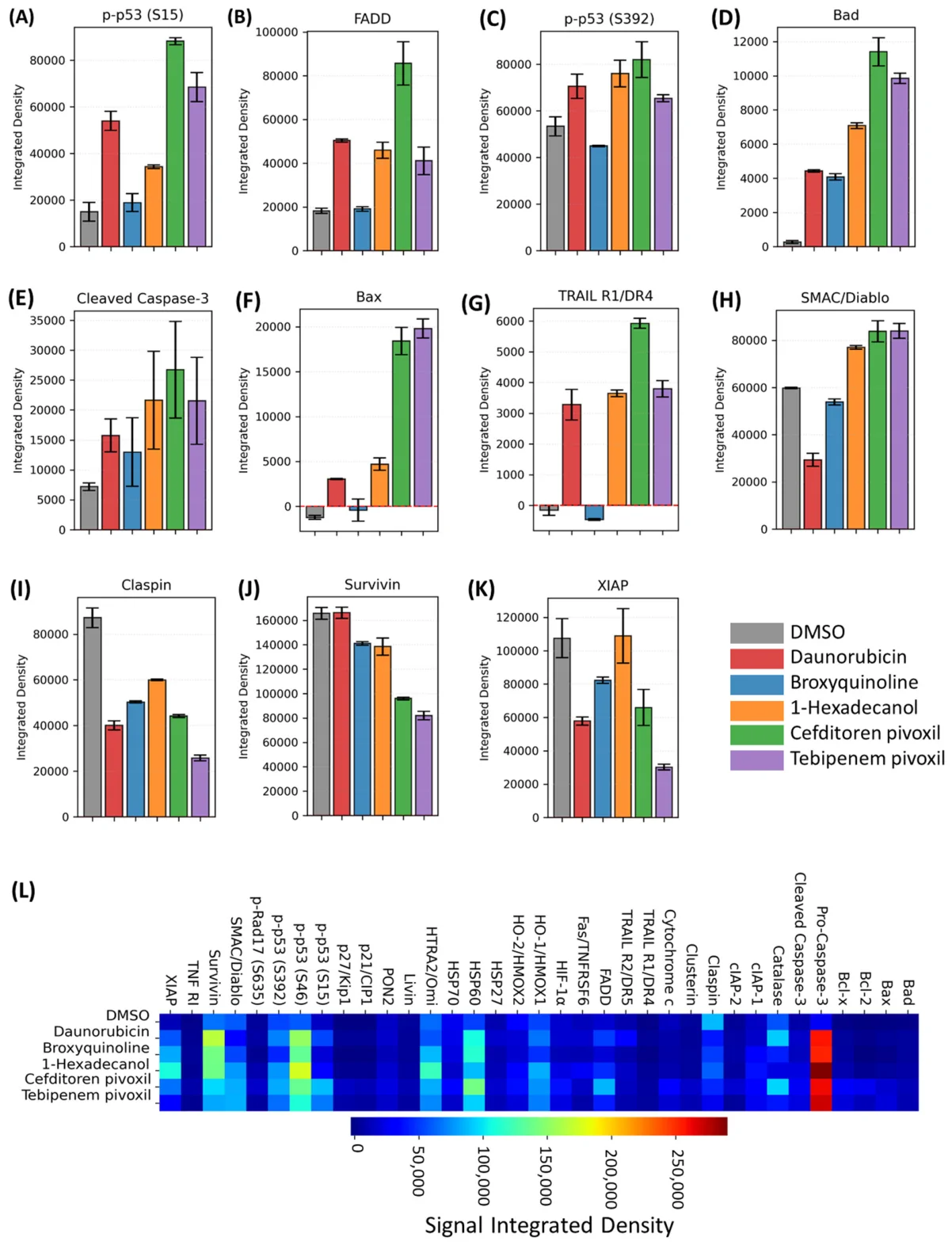
Drug-induced modulation of apoptosis-related proteins in KAIMRC-1 cells. (**A**–**K**) Bar graphs show background-corrected integrated density (mean ± SEM) for selected pro- and anti-apoptotic proteins measured by human apoptosis antibody array after 48 h treatment with DMSO, daunorubicin, broxyquinoline, hexadecanol, cefditoren pivoxil, or tebipenem pivoxil, including p-p53 (S15, S392), FADD, cleaved caspase-3, Bad, Bax, TRAIL R1/DR4, SMAC/Diablo, Claspin, XIAP, and survivin. (**L**) Heat map of log-scaled, background-corrected mean intensities for all arrayed proteins across the six conditions, with warmer colors indicating higher signal and highlighting coordinated induction of multiple pro-apoptotic markers, particularly in cefditoren pivoxil- and tebipenem pivoxil-treated cells, relative to more modest modulation of many anti-apoptotic proteins. Duplicate spot intensities for each protein were averaged per membrane. Mean and standard error of the mean (SEM) were computed across biological replicates using pandas group with mean and SEM aggregation functions, where SEM is calculated as SD/√n with Bessel’s correction (ddof = 1) applied to the standard deviation.

This phenomenon is consistent with stress-activated stabilization and transcriptional activation of p53 under these conditions and suggests that the β-lactam prodrugs may efficiently engage a p53-dependent damage response in KAIMRC-1 cells. Moreover, upstream adaptor FADD (Figure 8B) and the death receptor TRAIL R1/DR4 (Figure 8G) expression were also increased, mostly with cefditoren pivoxil and tebipenem pivoxil, suggesting that extrinsic receptor signaling contributes to the observed apoptotic response rather than only an intrinsic mitochondrial mechanism [54]. Mitochondrial pro-apoptotic markers also corresponded to this trend. Bax and Bad showed (Figure 8D,F) increased expression following exposure to hexadecanol, cefditoren pivoxil, and tebipenem pivoxil with respect to DMSO, in some cases with near-baseline or even slightly negative corrected values in the vehicle control (due to background subtraction) [55]. Similarly, Cleaved caspase-3, which plays a crucial role in apoptosis, was clearly induced across several treatments, with the largest increases under cefditoren pivoxil and hexadecanol and a substantial, though somewhat lower, induction by daunorubicin and tebipenem pivoxil [56]. Parallel elevation of SMAC/Diablo (Figure 8H) and cytochrome c further supports activation of mitochondrial outer membrane permeabilization and release of pro-apoptotic factors into the cytosol [57]. In contrast, multiple anti-apoptotic proteins like Bcl-2, Bcl-x, cIAP-1, cIAP-2, XIAP, and the chaperones HSP27/60/70 either remained relatively stable or showed less pronounced increase compared with the pro-apoptotic [58,59,60]. Notably, XIAP and survivin were moderately elevated, suggesting that the pro-apoptotic drive may exceed inhibitory buffering capacity under cefditoren pivoxil and tebipenem pivoxil exposure.

The heat map in Figure 8L provided a much broader view. Here, cefditoren pivoxil and tebipenem pivoxil generate the broadest “hot” signatures. Both drugs lead to the induction of p-p53 isoforms, FADD, TRAIL receptors, cleaved caspase-3, Bax, Bad, SMAC/Diablo, and several oxidative stress markers like HO-1/HMOX1, HIF-1α. Whereas, DMSO and broxyquinoline clustered toward relatively “cool” profiles. Daunorubicin and hexadecanol generated intermediate patterns, with substantial activation of selected mitochondrial and receptor-proximal proteins but less extensive network-wide upregulation. Together, these data indicate that the β-lactam prodrugs trigger a coordinated remodeling of the apoptotic landscape that integrates DNA-damage signaling, death receptor engagement, and mitochondrial amplification loops.

**Figure 9:**
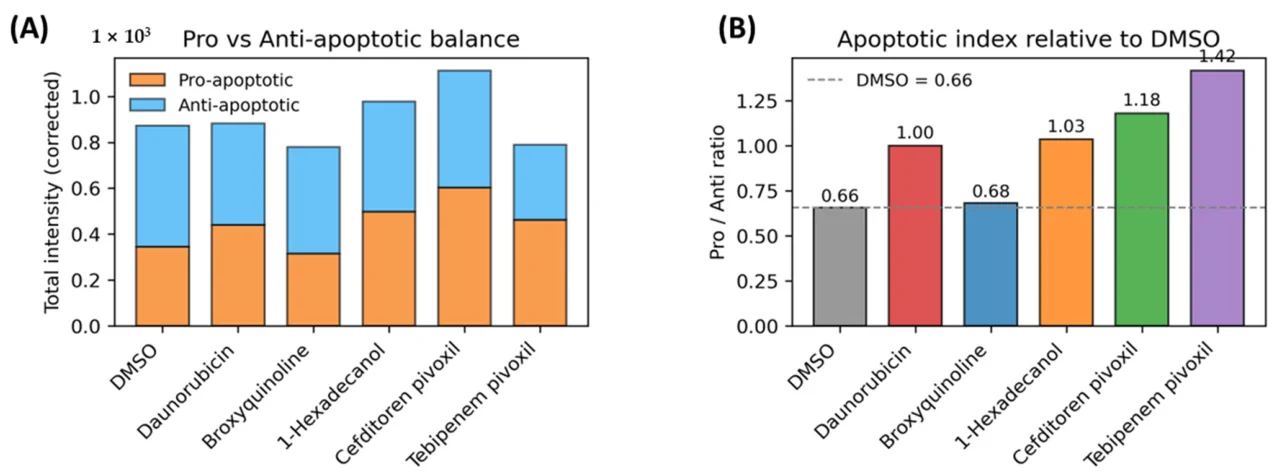
Global pro- versus anti-apoptotic balance and apoptotic index across treatments. (**A**) Stacked bar plot showing total background-corrected signal for pooled pro-apoptotic and anti-apoptotic markers, demonstrating that cefditoren pivoxil and tebipenem pivoxil markedly increase the pro-apoptotic pool compared with DMSO. (**B**) Apoptotic index (Pro/Anti ratio) for each treatment relative to the DMSO baseline (dashed line; ratio = 0.66).

The apoptotic index was defined as the ratio of summed background-corrected mean integrated density values across all pro-apoptotic proteins to the sum across all anti-apoptotic proteins captured on the array (Σ pro-apoptotic/Σ anti-apoptotic), providing a single composite metric of the net proteome-level balance between pro-death and pro-survival signalling across treatment conditions, conceptually grounded in the established Bcl-2 family rheostat model of apoptosis regulation [61,62]. Therefore, to quantify whether the net signalling balance favored cell death or survival, we aggregated corrected intensities into pro- and anti-apoptotic pools. The calculated apoptotic index for each treatment is shown in Figure 9A. In the DMSO control, the ratio of pro- to anti-apoptotic signal was <1 (0.66). This reflected a baseline dominance of survival factors consistent with a non-stressed state. Daunorubicin increased the pro-apoptotic pool and slightly reduced the anti-apoptotic sum, resulting in a global ratio normalized to ~1.0 (Figure 9B), indicative of a roughly balanced but primed apoptotic state. Hexadecanol and broxyquinoline produced only modest changes, with ratios of ~1.03 and ~0.68, respectively. Suggesting, that despite upregulation of individual markers, their overall impact on the apoptotic balance is limited in this system.

By contrast, cefditoren pivoxil and tebipenem pivoxil markedly shifted the total balance toward apoptosis. Cefditoren pivoxil increased the total pro-apoptotic signal to ~602,455 integrated units. While moderately raising anti-apoptotic proteins, yielding a Pro/Anti ratio of 1.18 (approximately 80% higher than DMSO). Tebipenem pivoxil further highlighted this skewing, with a high pro-apoptotic total (~462,292 units) and a relatively low anti-apoptotic total (~326,212 units), resulting in an apoptotic index of 1.42, more than two-fold above the DMSO reference. When plotted with treatment-specific colors, the apoptotic index panel highlights a clear rank order as Tebipenem pivoxil > cefditoren pivoxil ≫ daunorubicin ≈ hexadecanol > broxyquinoline ≳ DMSO in terms of net pro-apoptotic drive. These quantitative summaries are concordant with the protein-level heat map and bar graphs. This further reinforces the conclusion that cefditoren pivoxil and especially tebipenem pivoxil act as potent functional pro-apoptotic modulators in KAIMRC-1 cells, outperforming daunorubicin in shifting the integrated signalling landscape toward cell death.

## 4. Discussion

**Figure 10:**
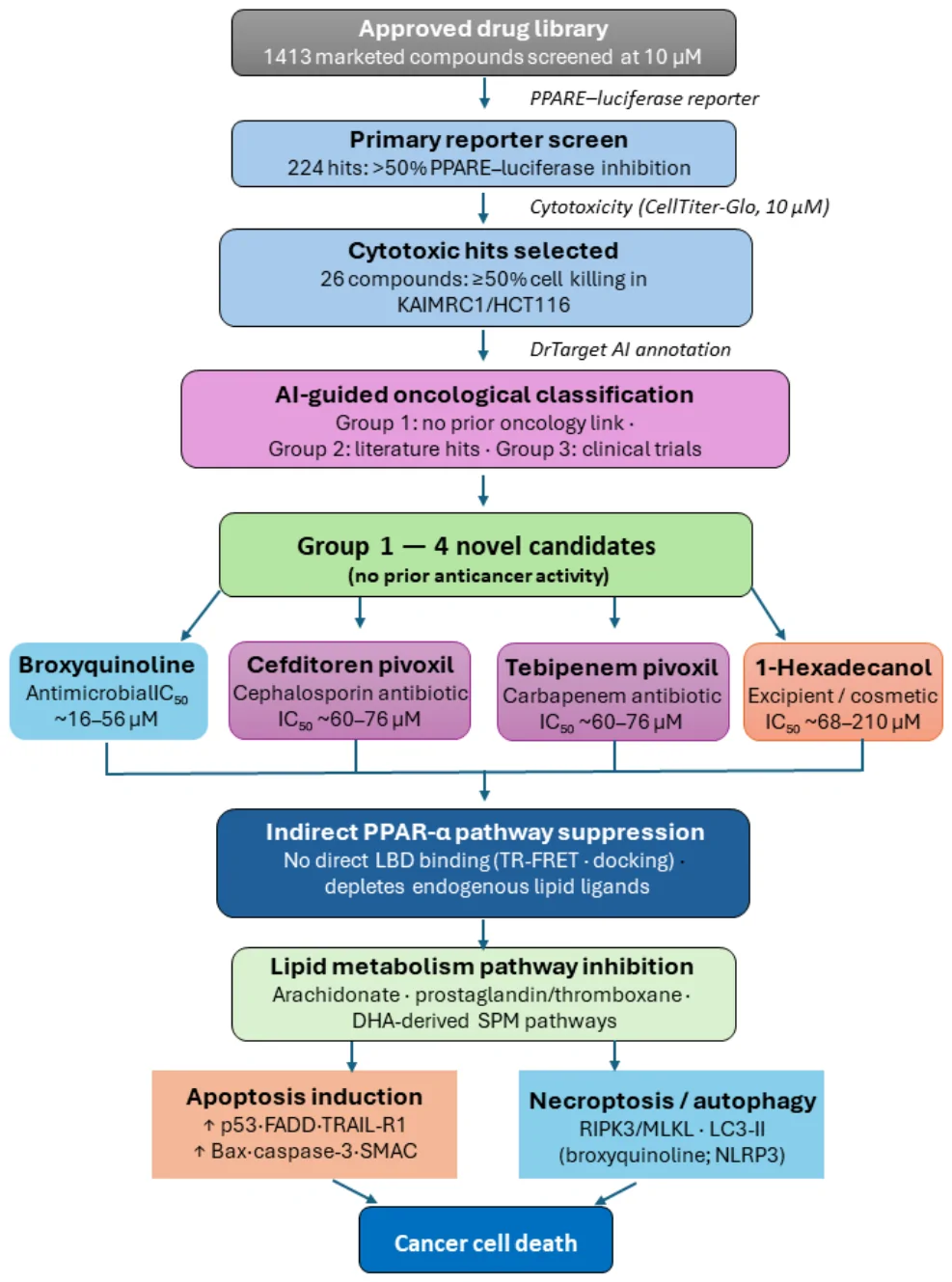
Flowchart representation of screening cascade. Compounds were screened at various stages to shortlist the most promising drug candidate showing anticancer activity under in vitro conditions.

In this study, we systematically screened a library of 1413 approved drugs, as shown in Figure 10. We looked for modulators of lipid metabolism with cytotoxic potential using a PPAR-α responsive PPARE–luciferase reporter in metabolically active cancer cell line models, KAIMRC1, HCT116, and SW480 (Figure 10). Our data confirm that KAIMRC1 and HCT116 have an active PPAR-α signaling, in line with reports that many epithelial tumors upregulate fatty-acid oxidation pathways to sustain growth under metabolic stress [19,24]. Exploiting this dependence, we identified 224 compounds that suppressed PPARE activity by more than 50%, suggesting specific interference with PPAR-α-driven transcriptional programs. Furthermore, only 26 of these reduced cell viability by at least 50% at 10 µM, indicating that pathway suppression is necessary but not sufficient for rapid cell death and that additional liabilities in bioenergetic or stress-response networks likely dictate cytotoxic outcome (Table 1).

AI-assisted annotation using the DrTarget platform enabled rational triaging of this large hit set. Compounds with established oncologic indications or extensive pre-existing cancer biology data were deprioritized. This allowed us to focus on four Group 1 molecules: cefditoren pivoxil, tebipenem pivoxil, 1-hexadecanol, and broxyquinoline. This type of computational curation is increasingly important in repurposing workflows. The DrTarget-predicted engagement of lipid metabolic routes such as arachidonate metabolism, prostaglandin and thromboxane biosynthesis, and DHA-derived SPM pathways. This provided a coherent mechanistic basis for the indirect suppression of PPARα-driven transcription we observe experimentally [40]. Depletion of these eicosanoid-class endogenous PPARα ligands would reduce constitutive receptor activation in KAIMRC1 cells without requiring direct LBD binding. This mechanism is analogous to the indirect PPARα modulation reported for certain fibrate-independent lipid-lowering agents [63]. The computational prediction of concurrent apoptotic pathway engagement, like caspase cascades, TNF signalling, and BH3 activation, was subsequently validated by the apoptosis proteomics data. The correlation of the data further lends credibility to the DrTarget pathway annotation as a predictive tool in repurposing workflows.

Notably, all four Group 1 candidates come from pharmacological classes not traditionally associated with cancer therapy. Two orally bioavailable β-lactam antibiotics, a long-chain fatty alcohol used largely as a formulation excipient, and a halogenated quinoline with mainly antimicrobial use. Dose–response data across cell lines revealed that these agents display reproducible but heterogeneous cytotoxicity. Daunorubicin behaved as expected, with low-micromolar IC_50_ values validating assay performance (Figure 3A,F). In contrast, cefditoren pivoxil, tebipenem pivoxil, and 1-hexadecanol showed moderate activity (IC_50_ ~60–210 µM) (Figure 3D–F), whereas broxyquinoline was clearly more potent (IC_50_ ~16–56 µM) (Figure 3C,F), particularly in SW480 cells. Quinoline scaffolds are increasingly recognized as privileged anticancer chemotypes capable of targeting DNA topology, kinases, and mitochondrial function, making broxyquinoline a plausible cytotoxic hit despite its non-oncologic provenance [48,49]. The ability of the β-lactam prodrugs and 1-hexadecanol to kill PPAR-competent cancer cells echoes reports that several anti-infectives and lipid-modifying agents can be repurposed as metabolic stressors or cell-cycle modulators in tumors.

The cytotoxic activity of cefditoren pivoxil and tebipenem pivoxil in cancer cells is consistent with an emerging body of literature linking β-lactam antibiotics to anticancer mechanisms beyond their classical bactericidal effects [64]. Several cephalosporins and carbapenems have been reported to interfere with mitochondrial function, generate reactive oxygen species, and disrupt membrane integrity in tumour cells [65,66]. A mechanistic basis for this off-target mitochondrial activity may reside in the evolutionary relationship between bacterial penicillin-binding proteins and LACTB. It is the only mammalian mitochondrial homolog of the bacterial PBP/β-lactamase superfamily, which functions as a tumour suppressor through regulation of mitochondrial phospholipid metabolism [67]. Pivalate released during ester hydrolysis depletes carnitine and may secondarily impair mitochondrial fatty-acid oxidation in cancer cells [68]. This carnitine-depleting mechanism may contribute to the moderate but reproducible cytotoxicity of these β-lactam prodrugs and is consistent with the indirect PPARα suppression observed in our reporter assays.

Mechanistically, our TR-FRET competitive binding experiments and molecular docking data consistently argue against these Group 1 compounds being classical PPAR-α ligands. Neither broxyquinoline nor 1-hexadecanol displaced the fluorescent tracer from the PPAR-α ligand-binding domain (LBD) (Figure 4). Docked poses also showed weak predicted affinity and no engagement of key antagonist residues such as Y314 or the displacement of helix 12 that characterizes GW6471 binding (Figure 6 and Figure 7). Cefditoren pivoxil and tebipenem pivoxil adopted conformations partially overlapping with the antagonist pocket but still lacked the critical Y314 interaction and scored far worse than GW6471 in docking. Together with the absence of tracer displacement in TR-FRET, these findings suggest that Group 1 compounds modulate PPAR-α-dependent transcription indirectly, potentially via effects on mitochondrial function, intracellular lipid pools, redox state, or upstream kinase pathways that feed into nuclear receptor co-regulator networks, as has been described for other PPAR modulators and fibrates.

Apoptosis-array profiling in KAIMRC1 cells provided further insight into the downstream consequences of treatment. As plotted in Figure 8, Cefditoren pivoxil and tebipenem pivoxil, in particular, induced a coordinated pro-apoptotic response encompassing phosphorylation of p53 at Ser15/Ser392 (Figure 8A,C), up-regulation of FADD and TRAIL-R1/DR4 (Figure 8B,G), activation of Bax/Bad, cytochrome c release, SMAC/Diablo elevation and robust cleavage of caspase-3 (Figure 8D–F,H,L). In contrast, multiple anti-apoptotic proteins (Bcl-2 family members, XIAP, survivin, cIAPs, and heat-shock proteins) were either modestly changed or increased to a lesser extent, yielding an apoptotic index (pro/anti ratio) substantially greater than that of both DMSO and the reference anthracycline. These data argue that the β-lactam prodrugs drive an integrated death-receptor and mitochondrial response that ultimately overwhelms intrinsic survival buffers. Hexadecanol and broxyquinoline produced weaker, more selective changes at the protein level, consistent with their lower apoptotic indices and intermediate IC_50_ values.

Among the Group 1 candidates, broxyquinoline merits particular mechanistic discussion. As a halogenated 8-hydroxyquinoline derivative, it is capable of chelating divalent metal ions, including zinc and copper, which are cofactors for multiple DNA-repair enzymes and proteasomal components. Metal chelation by quinoline derivatives has been linked to proteasome inhibition, topoisomerase interference, and mitochondrial membrane disruption in cancer cells [48]. Notably, broxyquinoline was recently identified as a direct NLRP3 inflammasome inhibitor targeting Arg165 of the NLRP3 protein, suggesting an additional inflammatory axis that may intersect with tumour-cell-intrinsic stress responses [69]. Its relatively modest effect on the apoptotic index in our array, despite low IC_50_ values, is consistent with a mechanism that differs from classical caspase activation—possibly involving necroptotic or autophagic cell death that the antibody array was not designed to capture.

An increasing number of chronically prescribed drugs are being evaluated for repurposing in oncology, reflecting the strong epidemiological and mechanistic links between metabolic, cardiovascular disease, and cancer. Among anti-diabetics, metformin is the most extensively studied; it activates AMPK, inhibits mTOR, and reduces insulin/IGF-1 signalling, and is currently in multiple Phase II–III trials as monotherapy or in combination regimens for breast, colorectal, prostate, lung, and endometrial cancers [69]. Other glucose-lowering agents such as pioglitazone and the SGLT2 inhibitor dapagliflozin are being tested in early-phase trials, exploiting their ability to modulate PPARγ activity, tumour metabolism, and the diabetic tumour microenvironment [70,71,72]. Repurposing efforts also extend to anti-hypertensives: non-selective β-blockers like propranolol and carvedilol, which dampen adrenergic signalling, angiogenesis, and stress-mediated immune evasion, are in Phase II studies across indications including breast, colorectal, pancreatic, and prostate cancer, either perioperatively or combined with standard chemotherapy [39,73,74]. In parallel, drugs targeting the renin–angiotensin system—ACE inhibitors (captopril, enalapril) and angiotensin receptor blockers (losartan, candesartan, irbesartan, telmisartan)—are being evaluated for their anti-angiogenic, anti-fibrotic, and pro-apoptotic properties in solid tumours, with particular interest in colorectal and gynaecologic malignancies [36,75,76,77,78]. In other studies, vasodilators such as hydralazine and minoxidil add a further layer, acting as epigenetic or metabolic modulators found to have promising results in cancer models [79,80]. Together, these examples illustrate how well-characterised cardiovascular and metabolic drugs are being strategically redeployed to exploit vulnerabilities in cancer growth, survival, and treatment resistance.

Our study demonstrates that integrating pathway-based luciferase screening with AI-guided annotation, orthogonal binding assays, in silico modelling, and apoptosis proteomics can uncover unconventional, repurposable molecules that perturb PPAR-linked metabolic survival circuits in cancer cells. In particular, broxyquinoline emerges as a promising quinoline lead with direct cytotoxicity, while cefditoren pivoxil and tebipenem pivoxil behave as potent functional pro-apoptotic modulators whose activity likely reflects complex crosstalk between antibiotic targets, metabolic stress, and PPAR-regulated lipid homeostasis.

## 5. Conclusions

Drug repurposing is a potential strategy for identifying anticancer candidates that already have an established safety profile and known pharmacokinetics. By exploiting constitutive PPAR-α activity as a functional screening entry point in breast and colorectal cancer cell models, we identified four non-oncologic compounds, namely broxyquinoline, 1-hexadecanol, cefditoren pivoxil, and tebipenem pivoxil. TR-FRET competitive binding and molecular docking data indicate that none engage the canonical PPAR-α LBD with significant affinity, implicating indirect modulation of the lipid metabolic and transcriptional axis rather than classical receptor antagonism. Broxyquinoline demonstrated the highest cytotoxic potency, while cefditoren pivoxil and tebipenem pivoxil produced the most extensive remodelling of the apoptotic signalling landscape. These findings demonstrate that systematic functional screening of approved drug libraries, combined with AI-guided annotation, can uncover unconventional repurposing candidates targeting metabolic vulnerabilities in solid tumours. The identified agents exhibit promising in vitro anticancer activity, and their established clinical safety profiles substantially lower the translational barrier relative to novel chemical entities. Pending validation in in vivo models, these compounds demonstrated a significant potential for repositioning as PPAR-α-pathway-targeted anticancer therapeutics.

## Figures and Tables

**Table 1:** Selected marketed drugs showing at least 50% inhibition of PPARE-Luciferase/PPAR-α activity in KAIMRC1 cell line were tested for the cell death assay. KAIMRC1 and HCT116 cells were treated with each compound at 10 µM for 48 h in triplicate, and cell death was measured using CellTiter-Glo to estimate IC50.

Group	Drug	Indication	Target	% Inhibition of PPAR-α Luciferase in KAIMRC1	SD % Inhibition of PPAR-α Luciferase in KAIMRC1	% KAIMRC1 Cell Killing	% HCT116 Cell Killing	SD % KAIMRC1 Killing	SD % HCT116 Killing
**Group 1 compounds:** No Past or ongoing clinical trials in cancer, and not known in the literature to kill cancer cells	1-Cefditoren Pivoxil	Infection	Penicillin-binding proteins	80	27	59	5	4	13
	2-Tebipenem Pivoxil	Infection	Carbapenem antibiotic	81	14	83	5	3	17
	3-1-Hexadecanol	Skin care	Not defined	59	10	61	0	6	19
	4-Broxyquinoline	Infection	NLR family pyrin domain-containing 3	88	26	81	86	2	11
**Group 2 compounds: ** No past or ongoing clinical trials in cancer, but it is known in the literature that it kills cancer cells	1-Etegravir	Immunology	Integrase	85	16	72	91	4	0
	2-Bendipine HCl	Cardiovascular Disease	Calcium Channel	73	11	91	97	1	0
	3-Flunarizine 2HCl	Neurological Disease	Calcium Channel	73	11	72	49	1	15
	4-Dibucaine HCl	Endocrinology	Sodium Channel	87	12	99	90	0	12
	5-Avanafil	Cardiovascular Disease	PDE	84	13	69	38	8	6
	6-Nifuroxazide	Infection	STAT3	75	23	91	99	1	0
	7-Chlorprothixene	Neurological Disease	Dopamine Receptor	47	10	100	97	0	1
	8-Cyandelate	Vasodilator medication	Others	80	23	53	25	6	5
	9-Procyclidine HCl	Parkinson Medication	NMDAR	74	11	77	34	5	2
	10-Manidipine	Cardiovascular Disease	Calcium Channel	58	14	74	70	1	1
**Group 3 compounds: ** There are ongoing clinical trials for cancer, or already marketed drugs against cancer	1-Riluzole	Neurological Disease	GluR, Sodium Channel	59	15	66	24	1	7
	2-Nitazoxanide	Infection	Others	81	18	75	17	1	7
	3-Dipyridamole	Cardiovascular Disease	PDE	90	14	83	47	2	6
	4-Lacidipine	Cardiovascular Disease	Calcium Channel	54	18	98	92	0	2
	5-Irinotecan HCl Trihydrate	Neurological Disease	Topoisomerase	85	13	82	61	4	2
	6-Trimebutine	Gastroenterology	Opioid Receptor	64	13	82	78	2	0
	7-Bexarotene	Cardiovascular Disease	Retinoid Receptor	54	12	39	30	3	11
	8-Atorvastatin Calcium	Cardiovascular Disease	HMG-CoA Reductase	54	12	60	26	8	5
	9-Fenbendazole	Infection/Parasitology	Parasite	58	10	91	84	2	4
	10-Alverine Citrate	Gastroenterology	OX Receptor	82	23	89	49	1	4
	11-Carfilzomib (PR-171)	Cardiovascular Disease	Proteasome	80	15	84	89	0	1
	12-Pentamidine Isethionate	Infection	Phosphatase	50	16	85	10	1	2

## Data Availability

Data will be made available on request.

## Supplementary Materials

The following supporting information can be downloaded at https://www.mdpi.com/article/10.3390/cancers18193231/s1, Figure S1: (A) Layout and coordinate map of the Human Apoptosis Antibody Array membrane (R&D Systems, Cat. #ARY009). The array membrane contains 35 duplicate antibody spots targeting distinct apoptosis-related proteins, arranged in a grid format across five row groups (A–E) and 24 column positions. Each protein target is represented by two adjacent replicate spots at the coordinates indicated in the table above. Positions A1–A2, A23–A24, and E1–E2 serve as internal reference spots used for membrane alignment and signal normalisation, while positions D23–D24 serve as negative controls (PBS). (B) Inverted image of blots. Signal intensities were quantified using ImageJ/Fiji as integrated density values following background subtraction.; Table S1: Coordinate reference map for the Human Apoptosis Antibody Array membrane (R&D Systems, Cat. #ARY009) used in this study. The table lists all 35 protein targets and control spots included on the array membrane, with their corresponding duplicate spot coordinates.; Table S2: DrTarget-predicted pathway engagement for Group 1 compounds (broxyquinoline, cefditoren pivoxil, tebipenem pivoxil, and 1-hexadecanol) based on ChEMBL-linked Reactome pathway annotation.

## Abbreviations

PPARs	Peroxisome Proliferator-Activated Receptors
RXRs	Retinoid X Receptors
PPREs	PPAR Response Elements
SMRT	Silencing Mediator for Retinoid and Thyroid Hormone Receptors
LBD	Ligand-Binding Domain
XP	Extra Precision
