# Supplementary material for: From Infection to Oncology: AI-Assisted Drug Repurposing Uncovers Anti-Infective and Excipient-Class Compounds as PPAR-α-Linked Anticancer Agents
Source: Cancers (Basel). 2026 Oct 8;18(19):3231. doi: 10.3390/cancers18193231 (PMC13664190; doi:10.3390/cancers18193231)
Supplement: Supplementary file 1 [file cancers-18-03231-s001.zip › cancers-4344463-supplementary.pdf]

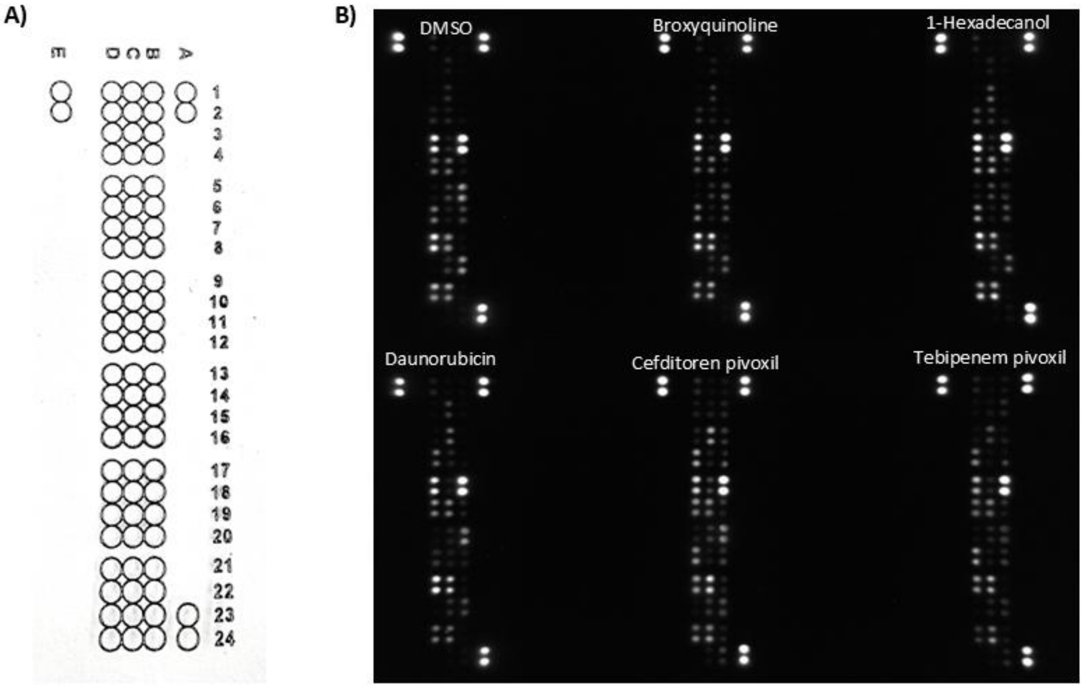

Figure S1: *A) Layout and coordinate map of the Human Apoptosis Antibody Array membrane (R&D Systems, Cat. #ARY009). The array membrane contains 35 duplicate antibody spots targeting distinct apoptosis-related proteins, arranged in a grid format across five row groups (A–E) and 24 column positions. Each protein target is represented by two adjacent replicate spots at the coordinates indicated in the table above. Positions A1–A2, A23–A24, and E1–E2 serve as internal reference spots used for membrane alignment and signal normalisation, while positions D23–D24 serve as negative controls (PBS). B) Inverted image of blots. Signal intensities were quantified using ImageJ/Fiji as integrated density values following background subtraction.*

Supplementary Table S1*: Coordinate reference map for the Human Apoptosis Antibody Array membrane (R&D Systems, Cat. #ARY009) used in this study. The table lists all 35 protein targets and control spots included on the array membrane, with their corresponding duplicate spot coordinates.*

	**Target / Control**	**Coordinate**	**Target / Control**
**A1, A2**	Reference spots	**C13, C14**	HO-2 / HMOX2
**A23, A24**	Reference spots	**C15, C16**	HSP27
**B1, B2**	Bad	**C17, C18**	HSP60
**B3, B4**	Bax	**C19, C20**	HSP70
**B5, B6**	Bcl-2	**C21, C22**	HTRA2 / Omi
**B7, B8**	Bcl-x	**C23, C24**	Livin
**B9, B10**	Pro-caspase-3	**D1, D2**	PON2
**B11, B12**	Cleaved caspase-3	**D3, D4**	p21 / CIP1 / CDKN1A
**B13, B14**	Catalase	**D5, D6**	p27 / Kip1
**B15, B16**	cIAP-1	**D7, D8**	Phospho-p53 (Ser15)
**B17, B18**	cIAP-2	**D9, D10**	Phospho-p53 (Ser46)
**B19, B20**	Claspin	**D11, D12**	Phospho-p53 (Ser392)
**B21, B22**	Clusterin	**D13, D14**	Phospho-Rad17 (Ser635)
**B23, B24**	Cytochrome c	**D15, D16**	SMAC / Diablo
**C1, C2**	TRAIL R1 / DR4	**D17, D18**	Survivin
**C3, C4**	TRAIL R2 / DR5	**D19, D20**	TNF RI / TNFRSF1A
**C5, C6**	FADD	**D21, D22**	XIAP
**C7, C8**	Fas / TNFRSF6 / CD95	**D23, D24**	PBS (negative control)
**C9, C10**	HIF-1α	**E1, E2**	Reference spots
**C11, C12**	HO-1 / HMOX1 / HSP32		

Supplementary Table S2: *DrTarget-predicted pathway engagement for Group 1 compounds (broxyquinoline, cefditoren pivoxil, tebipenem pivoxil, and 1-hexadecanol) based on ChEMBL-linked Reactome pathway annotation.*

Path Selection	PARENT_PATHWAY	PATHWAY_NAME	SUM SCORES	AVG SCORE
Apoptosis	Apoptosis	Apoptotic execution phase	4.65	4.65
Apoptosis	Apoptosis	Intrinsic Pathway for Apoptosis	5	5
Apoptosis	Apoptotic cleavage of cellular proteins	Caspase-mediated cleavage of cytoskeletal proteins	4.65	4.65
Apoptosis	Apoptotic execution phase	Apoptotic cleavage of cellular proteins	4.65	4.65
Apoptosis	Death Receptor Signaling	TNF signaling	10.3	5.15
Apoptosis	Disease	Diseases of programmed cell death	5	5
Apoptosis	Diseases of programmed cell death	Defective Intrinsic Pathway for Apoptosis	5	5
Apoptosis	Intrinsic Pathway for Apoptosis	Activation of BH3-only proteins	5	5
Apoptosis	Programmed Cell Death	Apoptosis	9.65	4.82
Apoptosis	Programmed Cell Death	Programmed Cell Death	15.8	5.27
Apoptosis	Programmed Cell Death	Regulated Necrosis	11.15	5.58
Apoptosis	Regulated Necrosis	Pyroptosis	5	5
Apoptosis	Regulated Necrosis	RIPK1-mediated regulated necrosis	6.15	6.15
Apoptosis	RIPK1-mediated regulated necrosis	Regulation of necroptotic cell death	6.15	6.15
Apoptosis	Signal Transduction	Death Receptor Signaling	10.3	5.15
Apoptosis	TNF signaling	Regulation of TNFR1 signaling	10.3	5.15
Apoptosis	TNF signaling	TNFR1-induced NF-kappa-B signaling pathway	10.3	5.15
Apoptosis	TNF signaling	TNFR1-induced proapoptotic signaling	10.3	5.15
Autophagy	Autophagy	Autophagy	10.47	5.24
Autophagy	Autophagy	Chaperone Mediated Autophagy	10.47	5.24
Autophagy	Autophagy	Late endosomal microautophagy	4.32	4.32
Autophagy	Autophagy	Macroautophagy	10.47	5.24
Autophagy	Macroautophagy	Selective autophagy	10.47	5.24
Cancer	Defective Intrinsic Pathway for Apoptosis	Loss of Function of TP53 in Cancer	5	5
Cancer	Deubiquitination	Ovarian tumor domain proteases	5	5

Cancer	Diseases of signal transduction by growth factor receptors and second messengers	Signaling by ALK in cancer	5	5
Cancer	Diseases of signal transduction by growth factor receptors and second messengers	Signaling by EGFR in Cancer	6.15	6.15
Cancer	Diseases of signal transduction by growth factor receptors and second messengers	Signaling by ERBB2 in Cancer	6.15	6.15
Cancer	Diseases of signal transduction by growth factor receptors and second messengers	Signaling by NOTCH1 in Cancer	4.32	4.32
Cancer	Diseases of signal transduction by growth factor receptors and second messengers	Signaling by TGF-beta Receptor Complex in Cancer	5.35	5.35
Cancer	Drug resistance in ERBB2 KD mutants	Resistance of ERBB2 KD mutants to AEE788	6.15	6.15
Cancer	Drug resistance in ERBB2 KD mutants	Resistance of ERBB2 KD mutants to afatinib	6.15	6.15
Cancer	Drug resistance in ERBB2 KD mutants	Resistance of ERBB2 KD mutants to lapatinib	6.15	6.15
Cancer	Drug resistance in ERBB2 KD mutants	Resistance of ERBB2 KD mutants to neratinib	6.15	6.15
Cancer	Drug resistance in ERBB2 KD mutants	Resistance of ERBB2 KD mutants to osimertinib	6.15	6.15
Cancer	Drug resistance in ERBB2 KD mutants	Resistance of ERBB2 KD mutants to sapitinib	6.15	6.15
Cancer	Drug resistance in ERBB2 KD mutants	Resistance of ERBB2 KD mutants to tesevatinib	6.15	6.15
Cancer	Drug resistance in ERBB2 KD mutants	Resistance of ERBB2 KD mutants to trastuzumab	6.15	6.15
Cancer	Loss of Function of SMAD2/3 in Cancer	SMAD2/3 MH2 Domain Mutants in Cancer	5.35	5.35
Cancer	Loss of Function of SMAD2/3 in Cancer	SMAD2/3 Phosphorylation Motif Mutants in Cancer	5.35	5.35
Cancer	Loss of Function of SMAD4 in Cancer	SMAD4 MH2 Domain Mutants in Cancer	5.35	5.35
Cancer	Loss of Function of TGFBR1 in Cancer	TGFBR1 KD Mutants in Cancer	5.35	5.35
Cancer	Loss of Function of TP53 in Cancer	Loss of function of TP53 in cancer due to loss of tetramerization ability	5	5

Cancer	Nuclear events mediated by NFE2L2	NFE2L2 regulating tumorigenic genes	4.54	4.54
Cancer	Oncogenic MAPK signaling	Signaling by BRAF and RAF1 fusions	6.45	6.45
Cancer	Signaling by EGFR in Cancer	Signaling by EGFRvIII in Cancer	6.15	6.15
Cancer	Signaling by EGFR in Cancer	Signaling by Ligand-Responsive EGFR Variants in Cancer	6.15	6.15
Cancer	Signaling by Ligand-Responsive EGFR Variants in Cancer	Constitutive Signaling by Ligand-Responsive EGFR Cancer Variants	6.15	6.15
Cancer	Signaling by NOTCH1 HD+PEST Domain Mutants in Cancer	Constitutive Signaling by NOTCH1 HD+PEST Domain Mutants	4.32	4.32
Cancer	Signaling by NOTCH1 in Cancer	Signaling by NOTCH1 HD+PEST Domain Mutants in Cancer	4.32	4.32
Cancer	Signaling by NOTCH1 in Cancer	Signaling by NOTCH1 PEST Domain Mutants in Cancer	4.32	4.32
Cancer	Signaling by NOTCH1 PEST Domain Mutants in Cancer	Constitutive Signaling by NOTCH1 PEST Domain Mutants	4.32	4.32
Cancer	Signaling by TGF-beta Receptor Complex in Cancer	Loss of Function of SMAD2/3 in Cancer	5.35	5.35
Cancer	Signaling by TGF-beta Receptor Complex in Cancer	Loss of Function of SMAD4 in Cancer	5.35	5.35
Cancer	Signaling by TGF-beta Receptor Complex in Cancer	Loss of Function of TGFBR1 in Cancer	5.35	5.35
Cancer Pathways	Cellular Senescence	Oncogene Induced Senescence	5	5
Cancer Pathways	G1/S DNA Damage Checkpoints	p53-Dependent G1/S DNA damage checkpoint	5	5
Cancer Pathways	Generic Transcription Pathway	Transcriptional Regulation by TP53	15.3	5.1
Cancer Pathways	p53-Dependent G1 DNA Damage Response	Stabilization of p53	5	5
Cancer Pathways	p53-Dependent G1 DNA Damage Response	Transcriptional activation of p53 responsive genes	5	5
Cancer Pathways	p53-Dependent G1/S DNA damage checkpoint	p53-Dependent G1 DNA Damage Response	5	5
Cancer Pathways	Regulation of TP53 Activity	Regulation of TP53 Activity through Acetylation	5	5
Cancer Pathways	Regulation of TP53 Activity	Regulation of TP53 Activity through Association with Co-factors	5	5
Cancer Pathways	Regulation of TP53 Activity	Regulation of TP53 Activity through Methylation	5	5

Cancer Pathways	Regulation of TP53 Activity	Regulation of TP53 Activity through Phosphorylation	5	5
Cancer Pathways	Regulation of TP53 Activity	Regulation of TP53 Expression and Degradation	15.3	5.1
Cancer Pathways	Regulation of TP53 Activity through Acetylation	PI5P Regulates TP53 Acetylation	5	5
Cancer Pathways	Regulation of TP53 Expression and Degradation	Regulation of TP53 Degradation	15.3	5.1
Cancer Pathways	Regulation of TP53 Expression and Degradation	Regulation of TP53 Expression	5	5
Cancer Pathways	Signaling by ALK in cancer	Signaling by ALK fusions and activated point mutants	5	5
Cancer Pathways	Signaling by EGFRvIII in Cancer	Constitutive Signaling by EGFRvIII	6.15	6.15
Cancer Pathways	Signaling by ERBB2	Downregulation of ERBB2 signaling	6.15	6.15
Cancer Pathways	Signaling by ERBB2	Drug-mediated inhibition of ERBB2 signaling	6.15	6.15
Cancer Pathways	Signaling by ERBB2 in Cancer	Constitutive Signaling by Overexpressed ERBB2	6.15	6.15
Cancer Pathways	Signaling by ERBB2 in Cancer	Drug resistance in ERBB2 KD mutants	6.15	6.15
Cancer Pathways	Signaling by ERBB2 in Cancer	Drug resistance in ERBB2 TMD/JMD mutants	6.15	6.15
Cancer Pathways	Signaling by ERBB2 in Cancer	Signaling by ERBB2 ECD mutants	6.15	6.15
Cancer Pathways	Signaling by ERBB2 in Cancer	Signaling by ERBB2 KD Mutants	6.15	6.15
Cancer Pathways	Signaling by ERBB2 in Cancer	Signaling by ERBB2 TMD/JMD mutants	6.15	6.15
Cancer Pathways	TP53 Regulates Transcription of Cell Cycle Genes	TP53 regulates transcription of additional cell cycle genes whose exact role in the p53 pathway remain uncertain	5	5
Cancer Pathways	TP53 Regulates Transcription of Cell Cycle Genes	TP53 Regulates Transcription of Genes Involved in G1 Cell Cycle Arrest	5	5
Cancer Pathways	TP53 Regulates Transcription of Cell Cycle Genes	TP53 Regulates Transcription of Genes Involved in G2 Cell Cycle Arrest	5	5
Cancer Pathways	TP53 Regulates Transcription of Cell Death Genes	TP53 Regulates Transcription of Caspase Activators and Caspases	5	5

Cancer Pathways	TP53 Regulates Transcription of Cell Death Genes	TP53 Regulates Transcription of Death Receptors and Ligands	5	5
Cancer Pathways	TP53 Regulates Transcription of Cell Death Genes	TP53 Regulates Transcription of Genes Involved in Cytochrome C Release	5	5
Cancer Pathways	TP53 Regulates Transcription of Cell Death Genes	TP53 regulates transcription of several additional cell death genes whose specific roles in p53-dependent apoptosis remain uncertain	5	5
Cancer Pathways	Transcriptional activation of p53 responsive genes	Transcriptional activation of cell cycle inhibitor p21	5	5
Cancer Pathways	Transcriptional Regulation by TP53	Regulation of TP53 Activity	15.3	5.1
Cancer Pathways	Transcriptional Regulation by TP53	TP53 Regulates Metabolic Genes	5	5
Cancer Pathways	Transcriptional Regulation by TP53	TP53 Regulates Transcription of Cell Cycle Genes	5	5
Cancer Pathways	Transcriptional Regulation by TP53	TP53 Regulates Transcription of Cell Death Genes	5	5
Cancer Pathways	Transcriptional Regulation by TP53	TP53 Regulates Transcription of DNA Repair Genes	5	5
Cell Cycle	Cell Cycle	Cell Cycle	16.57	5.52
Cell Cycle	Cell Cycle	Cell Cycle Checkpoints	5	5
Cell Cycle	Cell Cycle	Cell Cycle, Mitotic	16.57	5.52
Cell Cycle	Cell Cycle Checkpoints	G2/M Checkpoints	5	5
Cell Cycle	Cell Cycle, Mitotic	M Phase	6.15	6.15
Cell Cycle	Cell Cycle, Mitotic	Mitotic G1 phase and G1/S transition	5.42	5.42
Cell Cycle	Cell Cycle, Mitotic	Mitotic G2-G2/M phases	11.15	5.58
Cell Cycle	Cell Cycle, Mitotic	S Phase	5.42	5.42
Cell Cycle	Centrosome maturation	Loss of proteins required for interphase microtubule organization from the centrosome	6.15	6.15
Cell Cycle	Centrosome maturation	Recruitment of mitotic centrosome proteins and complexes	6.15	6.15
Cell Cycle	DNA Replication Pre-Initiation	Activation of the pre-replicative complex	5.42	5.42
Cell Cycle	DNA Replication Pre-Initiation	Assembly of the pre-replicative complex	5.42	5.42

Cell Cycle	G1/S Transition	Activation of the pre-replicative complex	5.42	5.42
Cell Cycle	G2/M Checkpoints	G2/M DNA damage checkpoint	5	5
Cell Cycle	G2/M Transition	AURKA Activation by TPX2	6.15	6.15
Cell Cycle	G2/M Transition	Centrosome maturation	6.15	6.15
Cell Cycle	G2/M Transition	Regulation of PLK1 Activity at G2/M Transition	6.15	6.15
Cell Cycle	G2/M Transition	The role of GTSE1 in G2/M progression after G2 checkpoint	11.15	5.58
Cell Cycle	Loss of proteins required for interphase microtubule organization from the centrosome	Loss of Nlp from mitotic centrosomes	6.15	6.15
Cell Cycle	M Phase	Mitotic Prometaphase	6.15	6.15
Cell Cycle	Mitotic G1 phase and G1/S transition	G1/S Transition	5.42	5.42
Cell Cycle	Mitotic G2-G2/M phases	G2/M Transition	11.15	5.58
Cell Cycle	Mitotic Prometaphase	Recruitment of NuMA to mitotic centrosomes	6.15	6.15
Cell Cycle	Synthesis of DNA	Switching of origins to a post-replicative state	5.42	5.42
DNA Repair	Cell Cycle Checkpoints	G1/S DNA Damage Checkpoints	5	5
DNA Repair	Cellular Senescence	DNA Damage/Telomere Stress Induced Senescence	5	5
DNA Repair	DNA Damage Bypass	Recognition of DNA damage by PCNA-containing replication complex	4.35	4.35
DNA Repair	DNA Damage/Telomere Stress Induced Senescence	Formation of Senescence-Associated Heterochromatin Foci (SAHF)	5	5
DNA Repair	DNA Double Strand Break Response	Recruitment and ATM-mediated phosphorylation of repair and signaling proteins at DNA double strand breaks	9.4	4.7
DNA Repair	DNA Double-Strand Break Repair	DNA Double Strand Break Response	9.4	4.7
DNA Repair	DNA Repair	Base Excision Repair	4.7	4.7
DNA Repair	DNA Repair	DNA Damage Bypass	8.9	4.45
DNA Repair	DNA Repair	DNA Double-Strand Break Repair	19.18	4.8
DNA Repair	DNA Repair	DNA Repair	28.23	4.71
DNA Repair	DNA Repair	Nucleotide Excision Repair	4.55	4.55

DNA Repair	DNA Replication	DNA Replication	5.42	5.42
DNA Repair	DNA Replication	DNA Replication Pre-Initiation	5.42	5.42
DNA Repair	DNA Replication	Synthesis of DNA	5.42	5.42
DNA Repair	Nucleotide Excision Repair	Global Genome Nucleotide Excision Repair (GG-NER)	4.55	4.55
DNA Repair	S Phase	Synthesis of DNA	5.42	5.42
Lipid Metabolism	Arachidonate metabolism	Synthesis of Prostaglandins (PG) and Thromboxanes (TX)	4.5	4.5
Lipid Metabolism	Biosynthesis of DHA-derived SPMs	Biosynthesis of D-series resolvins	4.5	4.5
Lipid Metabolism	Biosynthesis of DHA-derived SPMs	Biosynthesis of maresins	5.4	5.4
Lipid Metabolism	Biosynthesis of EPA-derived SPMs	Biosynthesis of E-series 18(S)-resolvins	4.5	4.5
Lipid Metabolism	Biosynthesis of maresins	Biosynthesis of maresin-like SPMs	5.4	5.4
Lipid Metabolism	Biosynthesis of specialized pro-resolving mediators (SPMs)	Biosynthesis of DHA-derived SPMs	9.9	4.95
Lipid Metabolism	Biosynthesis of specialized pro-resolving mediators (SPMs)	Biosynthesis of EPA-derived SPMs	4.5	4.5
Lipid Metabolism	Biosynthesis of specialized pro-resolving mediators (SPMs)	Biosynthesis of Lipoxins (LX)	4.5	4.5
Lipid Metabolism	Fatty acid metabolism	Arachidonate metabolism	4.5	4.5
Lipid Metabolism	Metabolism	Metabolism of lipids	35.9	5.13
Lipid Metabolism	Metabolism of lipids	Biosynthesis of specialized pro-resolving mediators (SPMs)	9.9	4.95
Lipid Metabolism	Metabolism of lipids	Fatty acid metabolism	4.5	4.5
